# Supplementary material for: Effect of Gut Microbiota Alteration on Colorectal Cancer Progression in an In Vivo Model: Histopathological and Immunological Evaluation
Source: Curr Issues Mol Biol. 2025 Dec 23;48(1):15. doi: 10.3390/cimb48010015 (PMC12839836; doi:10.3390/cimb48010015)
Supplement: Supplementary file 1 [file cimb-48-00015-s001.zip › cimb-4035328-supplementary.pdf]

S1: informed consent

Anexo. Consentimiento informado pacientes

Proyecto:

El presente documento pretende obtener su declaración voluntaria de participación en el proyecto de investigación, cuyo propósito es la caracterización de la micobiota en pacientes con cáncer colo rectal y su posible asociación con factores clínicos.
El proyecto será realizado por el grupo de investigación de la Universidad de Santander y el grupo de Micología Médica y Experimental de la Corporación para Investigaciones (CIB-UDES)..

Para desarrollar el proyecto, se requerirá de su parte:
- muestras seriadas de materia fecal
- Autorización para usar el aislamiento fúngico obtenido a partir de la muestra clínica para su secuenciación y análisis metagenómico.
- Autorización para el acceso y uso de los siguientes datos presentes en su historia clínica, incluyendo entre otros: sexo, edad, resultado final de diagnóstico,estudios imagenologicos previos, paraclinicos y estudios anatomopatologicos, ademas de esquema de tratamientos oncologicos utilizados.

Este procedimiento será realizado por un profesional (Médico, Enfermero o Microbiólogo), la toma de muestra no implica daño a su integridad y no suele generar malestar.Una vez obtenido el resultado los datos serán confidenciales cumpliendo la ley 1581 de 2012: *habeas data*

Los resultados derivados del proyecto tendrán un uso exclusivo de investigación.

Su participación en el proyecto no le generará un beneficio económico o de manejo en el hospital, sin embargo, con los resultados de esta investigación se espera generar más evidencia sobre la situación actual de la relación de la micobiota y el cáncer colorrectal.

Finalmente, los investigadores del proyecto se comprometen a realizar una socialización de resultados con todos los participantes, una vez finalizada la investigación.

Basados en lo anterior,

Yo____,
identificado(a) con cédula de ciudadanía №_________________, por medio de este documento manifiesto que:

1. He sido invitado a participar voluntariamente en esta investigación aportando las muestras de materia fecal requeridas y autorizando el uso de ellas con fines de investigación y autorizando el uso de la información presente en mi historia clínica.
2. He sido informado(a) acerca del proyecto, y que la toma de estas muestras sólo

implica un riesgo mínimo o nulo para la salud.

3. Acepto que no recibirá compensación alguna y que liberó a los investigadores e instituciones participantes de toda obligación económica para conmigo.
4. Se me ha explicado que los procedimientos de análisis a los que se sometan las muestras no tendrán ningún costo para mí.
5. Se me ha asegurado que los resultados obtenidos a partir de la muestra se manejan de manera responsable y sólo con fines académicos y científicos.
6. Se me ha informado que se protegerá mi privacidad, a través de la reserva de mi identidad, omitiendo cualquier dato que permita identificarte en el momento de la divulgación de los resultados de la investigación. Todos los datos e información recolectada serán protegidos bajo la ley 1581 de 2012. Además, la historia clínica será custodiada bajo normativas propias institucionales y siguiendo la Resolución 839 de 2017.
7. Se me ha explicado que, en cualquier momento, en caso de alguna duda o inconveniente relacionado con la investigación, podré contactar a Dr Oscar Eduardo Roa Acosta, miembro del Grupo de Investigación Médica y Experimental al teléfono celular 3122811338
8. Entiendo que mi participación en esta investigación puede terminar en el momento en que lo decida y lo exprese a los investigadores responsables sin que perjudique mi futura atención. Soy libre de retirarme en cualquier momento.
9. Mi firma en este documento manifiesta mi participación voluntaria en este proyecto de investigación.
10. Las muestras de materia fecal sólo serán utilizadas con fines de investigación y una vez finalizada la misma, serán descartadas.
11. Se me ha informado que en caso de ser requerido, se me brindará un apoyo por un valor de $30.000, por concepto de transporte para la entrega de muestras de materia fecal.

*TODO PACIENTE QUEDA CON COPIA DEL CONSENTIMIENTO.

El presente consentimiento se firma en Santiago de Cali a los ___ días del mes de __________ del año _________.

____

Firma
Nombre Participante:
c.c.:
Dirección:
Teléfono:

____

Firma
Nombre Testigo 1:
c.c.:
Dirección:
Teléfono:

S2: Fecal Donor Questionnaire

Formulario Donantes de Material Fecal

Verificación de criterios de inclusión para la selección de heces de voluntarios aparentemente sanos.

Proyecto: *Evaluación preclínica del efecto de la modulación de la microbiota fúngica sobre la quimioterapia del cáncer colorrectal.*

Información general

Número del registro:			Fecha (D/M/A):
Nombre:			
Sexo:	Femenino	Masculino	Profesión:
Fecha de nacimiento (D/M/A):			Edad:
Peso corporal (Kg):			Talla (m):
Teléfono:			e- mail:

Lista de chequeo

Por favor responda **Si** o **No** a la siguiente lista marcando con un x la casilla que corresponda:

	**Si**	**No**
1. Es fumador		
2. Tiene alguna enfermedad gastrointestinal como gastritis, ulcera gástrica, colitis, colitis ulcerativa, enfermedad de Crohn, colon irritable, entre otros.		
3. Tiene o ha tenido en los últimos dos meses alguna enfermedad infecciosa como gastroenteritis, neumonía, tuberculosis, infección urinaria, parasitosis intestinal, entre otros.		
4. Tiene o ha tenido alguna enfermedad neoplásica o cáncer.		
5. Tiene alguna enfermedad autoinmune como artritis reumatoide, lupus, entre otras.		
6. Tiene alguna enfermedad metabólica como diabetes, dislipidemias, hipertensión arterial, etc.		
7. Sufre de estreñimiento o diarrea.		

8. Ha tenido diarrea aguda en el último mes.		
9. Es intolerante a algún alimento como el gluten, la lactosa, entre otras.		
10. Tiene alguna alergia alimentaria.		
11. Sufre o ha sufrido en los últimos meses de depresión, ansiedad o alguna enfermedad neurológica.		
12. Tiene un nivel de estrés alto en este momento o lo ha tenido en los últimos dos meses.		
13. Ha consumido antiinflamatorios, antibióticos o antiparasitarios en los últimos tres meses.		
14. Consume algún otro medicamento con regularidad.		
15. Ha consumido antibióticos y/o antiparasitarios en las últimas 4 semanas.		
16. Consume alcohol de forma frecuente.		
17. Tiene o ha seguido tratamientos para la pérdida de peso.		
18. Ha consumido laxantes en los últimos dos meses.		
19. Ha consumido suplementos nutricionales en el último mes.		
20. Tiene una dieta vegetariana o vegana.		
21. Consume carnes rojas o procesadas más de tres veces a la semana.		
22. Consume frutas y verduras todos los días.		
23. Realiza ejercicio de forma regular al menos 30 minutos diarios o su equivalencia.		
24. Es un(a) deportista de alto rendimiento.		
25. Ha viajado en los últimos 3 meses a una zona de alta incidencia de enfermedades tropicales como malaria, fiebre amarilla, etc.		
26. Ha estado hospitalizado en los últimos 6 meses.		
27. Le han realizado algún procedimiento quirúrgico en los últimos 3 meses.		

______________________________ ______________________________

Firma del donante Firma del investigador

Supplementary material

S3:

Table Analyzed	Histopathology of distal colonic tissue week 5				
Two-way ANOVA	Ordinary				
Alpha	0,05				

Source of Variation	% of total variation	P value	P value summary	Significant?
Interaction	13,95	0,0007	***	Yes
Row Factor	61,9	<0,0001	****	Yes
Column Factor	1,098	0,0132	*	Yes

ANOVA table	SS	DF	MS	F (DFn, DFd)	P value
Interaction	19,78	60	0,3296	F (60, 186) = 1,877	P=0,0007
Row Factor	87,73	30	2,924	F (30, 186) = 16,65	P<0,0001
Column Factor	1,556	2	0,7778	F (2, 186) = 4,429	P=0,0132
Residual	32,67	186	0,1756		

Within each row, compare columns (simple effects within rows)

Number of families	31
Number of comparisons per family	3
Alpha	0,05

Tukey's multiple comparisons test	Mean Diff,	95,00% CI of diff,	Significant?	Summary	Adjusted P Value
Epithelial atrophy					
Positive Control vs. FMT-CCR	0	-0,8084 to 0,8084	No	ns	>0,9999
Positive Control vs. FMT-Healthy	0	-0,8084 to 0,8084	No	ns	>0,9999
FMT-CCR vs. FMT-Healthy	0	-0,8084 to 0,8084	No	ns	>0,9999
Glandular atrophy					
Positive Control vs. FMT-CCR	0	-0,8084 to 0,8084	No	ns	>0,9999
Positive Control vs. FMT-Healthy	-0,3333	-1,142 to 0,4751	No	ns	0,5941
FMT-CCR vs. FMT-Healthy	-0,3333	-1,142 to 0,4751	No	ns	0,5941
Goblet cell					
Positive Control vs. FMT-CCR	0	-0,8084 to 0,8084	No	ns	>0,9999
Positive Control vs. FMT-Healthy	-0,3333	-1,142 to 0,4751	No	ns	0,5941
FMT-CCR vs. FMT-Healthy	-0,3333	-1,142 to 0,4751	No	ns	0,5941

Epithelial hyperplasia					
Positive Control vs. FMT-CCR	0	-0,8084 to 0,8084	No	ns	>0,9999
Positive Control vs. FMT-Healthy	0,3333	-0,4751 to 1,142	No	ns	0,5941
FMT-CCR vs. FMT-Healthy	0,3333	-0,4751 to 1,142	No	ns	0,5941
Glandular hyperplasia					
Positive Control vs. FMT-CCR	0	-0,8084 to 0,8084	No	ns	>0,9999
Positive Control vs. FMT-Healthy	0	-0,8084 to 0,8084	No	ns	>0,9999
FMT-CCR vs. FMT-Healthy	0	-0,8084 to 0,8084	No	ns	>0,9999
Mitosis					
Positive Control vs. FMT-CCR	-1,333	-2,142 to -0,5249	Yes	***	0,0004
Positive Control vs. FMT-Healthy	0	-0,8084 to 0,8084	No	ns	>0,9999
FMT-CCR vs. FMT-Healthy	1,333	0,5249 to 2,142	Yes	***	0,0004
Apoptosis					
Positive Control vs. FMT-CCR	-0,3333	-1,142 to 0,4751	No	ns	0,5941
Positive Control vs. FMT-Healthy	-0,3333	-1,142 to 0,4751	No	ns	0,5941
FMT-CCR vs. FMT-Healthy	0	-0,8084 to 0,8084	No	ns	>0,9999
Aberrant crypts					
Positive Control vs. FMT-CCR	-0,6667	-1,475 to 0,1418	No	ns	0,1282
Positive Control vs. FMT-Healthy	-0,6667	-1,475 to 0,1418	No	ns	0,1282
FMT-CCR vs. FMT-Healthy	0	-0,8084 to 0,8084	No	ns	>0,9999
Epithelial dysplasia					
Positive Control vs. FMT-CCR	-0,3333	-1,142 to 0,4751	No	ns	0,5941
Positive Control vs. FMT-Healthy	0	-0,8084 to 0,8084	No	ns	>0,9999
FMT-CCR vs. FMT-Healthy	0,3333	-0,4751 to 1,142	No	ns	0,5941
Glandular dysplasia					
Positive Control vs. FMT-CCR	-0,6667	-1,475 to 0,1418	No	ns	0,1282
Positive Control vs. FMT-Healthy	-1	-1,808 to -0,1916	Yes	*	0,0108
FMT-CCR vs. FMT-Healthy	-0,3333	-1,142 to 0,4751	No	ns	0,5941
Neoplastic proliferation					
Positive Control vs. FMT-CCR	-1,667	-2,475 to -0,8582	Yes	****	<0,0001
Positive Control vs. FMT-Healthy	-0,6667	-1,475 to 0,1418	No	ns	0,1282
FMT-CCR vs. FMT-Healthy	1	0,1916 to 1,808	Yes	*	0,0108
Neoplastic infiltration					

Positive Control vs. FMT-CCR	-0,3333	-1,142 to 0,4751	No	ns	0,5941
Positive Control vs. FMT-Healthy	-0,3333	-1,142 to 0,4751	No	ns	0,5941
FMT-CCR vs. FMT-Healthy	0	-0,8084 to 0,8084	No	ns	>0,9999
GALT hyperplasia					
Positive Control vs. FMT-CCR	0,3333	-0,4751 to 1,142	No	ns	0,5941
Positive Control vs. FMT-Healthy	0,6667	-0,1418 to 1,475	No	ns	0,1282
FMT-CCR vs. FMT-Healthy	0,3333	-0,4751 to 1,142	No	ns	0,5941
Erosion					
Positive Control vs. FMT-CCR	0,3333	-0,4751 to 1,142	No	ns	0,5941
Positive Control vs. FMT-Healthy	0,3333	-0,4751 to 1,142	No	ns	0,5941
FMT-CCR vs. FMT-Healthy	-2,22E-16	-0,8084 to 0,8084	No	ns	>0,9999
Ulceration					
Positive Control vs. FMT-CCR	0	-0,8084 to 0,8084	No	ns	>0,9999
Positive Control vs. FMT-Healthy	0	-0,8084 to 0,8084	No	ns	>0,9999
FMT-CCR vs. FMT-Healthy	0	-0,8084 to 0,8084	No	ns	>0,9999
Pigment					
Positive Control vs. FMT-CCR	0	-0,8084 to 0,8084	No	ns	>0,9999
Positive Control vs. FMT-Healthy	0	-0,8084 to 0,8084	No	ns	>0,9999
FMT-CCR vs. FMT-Healthy	0	-0,8084 to 0,8084	No	ns	>0,9999
Minerals					
Positive Control vs. FMT-CCR	0	-0,8084 to 0,8084	No	ns	>0,9999
Positive Control vs. FMT-Healthy	0	-0,8084 to 0,8084	No	ns	>0,9999
FMT-CCR vs. FMT-Healthy	0	-0,8084 to 0,8084	No	ns	>0,9999
Necrosis					
Positive Control vs. FMT-CCR	-0,6667	-1,475 to 0,1418	No	ns	0,1282
Positive Control vs. FMT-Healthy	0,3333	-0,4751 to 1,142	No	ns	0,5941
FMT-CCR vs. FMT-Healthy	1	0,1916 to 1,808	Yes	*	0,0108
Congestion					
Positive Control vs. FMT-CCR	-0,6667	-1,475 to 0,1418	No	ns	0,1282
Positive Control vs. FMT-Healthy	-1	-1,808 to -0,1916	Yes	*	0,0108
FMT-CCR vs. FMT-Healthy	-0,3333	-1,142 to 0,4751	No	ns	0,5941
Edema					
Positive Control vs. FMT-CCR	4,441e-016	-0,8084 to 0,8084	No	ns	>0,9999
Positive Control vs. FMT-Healthy	-0,3333	-1,142 to 0,4751	No	ns	0,5941

FMT-CCR vs. FMT-Healthy		-0,3333	-1,142 to 0,4751	No	ns	0,5941
Hemorrhage						
Positive Control vs. FMT-CCR	3,331e-016		-0,8084 to 0,8084	No	ns	>0,9999
Positive Control vs. FMT-Healthy		0	-0,8084 to 0,8084	No	ns	>0,9999
FMT-CCR vs. FMT-Healthy		-3,33E-16	-0,8084 to 0,8084	No	ns	>0,9999
Thrombosis						
Positive Control vs. FMT-CCR	3,331e-016		-0,8084 to 0,8084	No	ns	>0,9999
Positive Control vs. FMT-Healthy		0	-0,8084 to 0,8084	No	ns	>0,9999
FMT-CCR vs. FMT-Healthy		-3,33E-16	-0,8084 to 0,8084	No	ns	>0,9999
Fibrin						
Positive Control vs. FMT-CCR	2,220e-016		-0,8084 to 0,8084	No	ns	>0,9999
Positive Control vs. FMT-Healthy		0	-0,8084 to 0,8084	No	ns	>0,9999
FMT-CCR vs. FMT-Healthy		-2,22E-16	-0,8084 to 0,8084	No	ns	>0,9999
Exocytosis						
Positive Control vs. FMT-CCR	2,220e-016		-0,8084 to 0,8084	No	ns	>0,9999
Positive Control vs. FMT-Healthy		0	-0,8084 to 0,8084	No	ns	>0,9999
FMT-CCR vs. FMT-Healthy		-2,22E-16	-0,8084 to 0,8084	No	ns	>0,9999
Neutrophils						
Positive Control vs. FMT-CCR		-0,3333	-1,142 to 0,4751	No	ns	0,5941
Positive Control vs. FMT-Healthy		-0,3333	-1,142 to 0,4751	No	ns	0,5941
FMT-CCR vs. FMT-Healthy		0	-0,8084 to 0,8084	No	ns	>0,9999
Eosinophils						
Positive Control vs. FMT-CCR	3,331e-016		-0,8084 to 0,8084	No	ns	>0,9999
Positive Control vs. FMT-Healthy		0	-0,8084 to 0,8084	No	ns	>0,9999
FMT-CCR vs. FMT-Healthy		-3,33E-16	-0,8084 to 0,8084	No	ns	>0,9999
Macrophages						
Positive Control vs. FMT-CCR		-0,3333	-1,142 to 0,4751	No	ns	0,5941
Positive Control vs. FMT-Healthy		0,3333	-0,4751 to 1,142	No	ns	0,5941
FMT-CCR vs. FMT-Healthy		0,6667	-0,1418 to 1,475	No	ns	0,1282
Lymphocytes						
Positive Control vs. FMT-CCR		0	-0,8084 to 0,8084	No	ns	>0,9999
Positive Control vs. FMT-Healthy		0,6667	-0,1418 to 1,475	No	ns	0,1282
FMT-CCR vs. FMT-Healthy		0,6667	-0,1418 to 1,475	No	ns	0,1282

Plasma cells					
Positive Control vs. FMT-CCR	0	-0,8084 to 0,8084	No	ns	>0,9999
Positive Control vs. FMT-Healthy	0,3333	-0,4751 to 1,142	No	ns	0,5941
FMT-CCR vs. FMT-Healthy	0,3333	-0,4751 to 1,142	No	ns	0,5941
Fibrosis					
Positive Control vs. FMT-CCR	1,333	0,5249 to 2,142	Yes	***	0,0004
Positive Control vs. FMT-Healthy	1,333	0,5249 to 2,142	Yes	***	0,0004
FMT-CCR vs. FMT-Healthy	-8,88E-16	-0,8084 to 0,8084	No	ns	>0,9999
Biological agents					
Positive Control vs. FMT-CCR	0	-0,8084 to 0,8084	No	ns	>0,9999
Positive Control vs. FMT-Healthy	0	-0,8084 to 0,8084	No	ns	>0,9999
FMT-CCR vs. FMT-Healthy	0	-0,8084 to 0,8084	No	ns	>0,9999

Test details	Mean 1	Mean 2	Mean Diff,	SE of diff,	N1	N2	q	DF
Epithelial atrophy								
Positive Control vs. FMT-CCR	0	0	0	0,3422	3	3	0	186
Positive Control vs. FMT-Healthy	0	0	0	0,3422	3	3	0	186
FMT-CCR vs. FMT-Healthy	0	0	0	0,3422	3	3	0	186
Glandular atrophy								
Positive Control vs. FMT-CCR	0	0	0	0,3422	3	3	0	186
Positive Control vs. FMT-Healthy	0	0,3333	-0,3333	0,3422	3	3	1,378	186
FMT-CCR vs. FMT-Healthy	0	0,3333	-0,3333	0,3422	3	3	1,378	186
Goblet cell								
Positive Control vs. FMT-CCR	0	0	0	0,3422	3	3	0	186
Positive Control vs. FMT-Healthy	0	0,3333	-0,3333	0,3422	3	3	1,378	186
FMT-CCR vs. FMT-Healthy	0	0,3333	-0,3333	0,3422	3	3	1,378	186
Epithelial hyperplasia								
Positive Control vs. FMT-CCR	0,3333	0,3333	0	0,3422	3	3	0	186
Positive Control vs. FMT-Healthy	0,3333	0	0,3333	0,3422	3	3	1,378	186
FMT-CCR vs. FMT-Healthy	0,3333	0	0,3333	0,3422	3	3	1,378	186
Glandular hyperplasia								
Positive Control vs. FMT-CCR	0,6667	0,6667	0	0,3422	3	3	0	186
Positive Control vs. FMT-Healthy	0,6667	0,6667	0	0,3422	3	3	0	186
FMT-CCR vs. FMT-Healthy	0,6667	0,6667	0	0,3422	3	3	0	186

Mitosis									
Positive Control vs. FMT-CCR		1	2,333	-1,333	0,3422	3	3	5,511	186
Positive Control vs. FMT-Healthy		1	1	0	0,3422	3	3	0	186
FMT-CCR vs. FMT-Healthy		2,333	1	1,333	0,3422	3	3	5,511	186
Apoptosis									
Positive Control vs. FMT-CCR		0	0,3333	-0,3333	0,3422	3	3	1,378	186
Positive Control vs. FMT-Healthy		0	0,3333	-0,3333	0,3422	3	3	1,378	186
FMT-CCR vs. FMT-Healthy		0,3333	0,3333	0	0,3422	3	3	0	186
Aberrant crypts									
Positive Control vs. FMT-CCR		0	0,6667	-0,6667	0,3422	3	3	2,755	186
Positive Control vs. FMT-Healthy		0	0,6667	-0,6667	0,3422	3	3	2,755	186
FMT-CCR vs. FMT-Healthy		0,6667	0,6667	0	0,3422	3	3	0	186
Epithelial dysplasia									
Positive Control vs. FMT-CCR		0	0,3333	-0,3333	0,3422	3	3	1,378	186
Positive Control vs. FMT-Healthy		0	0	0	0,3422	3	3	0	186
FMT-CCR vs. FMT-Healthy		0,3333	0	0,3333	0,3422	3	3	1,378	186
Glandular dysplasia									
Positive Control vs. FMT-CCR	2,220e-016		0,6667	-0,6667	0,3422	3	3	2,755	186
Positive Control vs. FMT-Healthy	2,220e-016		1	-1	0,3422	3	3	4,133	186
FMT-CCR vs. FMT-Healthy		0,6667	1	-0,3333	0,3422	3	3	1,378	186
Neoplastic proliferation									
Positive Control vs. FMT-CCR		1	2,667	-1,667	0,3422	3	3	6,888	186
Positive Control vs. FMT-Healthy		1	1,667	-0,6667	0,3422	3	3	2,755	186
FMT-CCR vs. FMT-Healthy		2,667	1,667	1	0,3422	3	3	4,133	186
Neoplastic infiltration									
Positive Control vs. FMT-CCR		0,6667	1	-0,3333	0,3422	3	3	1,378	186
Positive Control vs. FMT-Healthy		0,6667	1	-0,3333	0,3422	3	3	1,378	186
FMT-CCR vs. FMT-Healthy		1	1	0	0,3422	3	3	0	186
GALT hyperplasia									
Positive Control vs. FMT-CCR		1,667	1,333	0,3333	0,3422	3	3	1,378	186
Positive Control vs. FMT-Healthy		1,667	1	0,6667	0,3422	3	3	2,755	186
FMT-CCR vs. FMT-Healthy		1,333	1	0,3333	0,3422	3	3	1,378	186
Erosion									

Positive Control vs. FMT-CCR		0,3333	-2,22E-16		0,3333	0,3422	3	3	1,378	186
Positive Control vs. FMT-Healthy		0,3333	0		0,3333	0,3422	3	3	1,378	186
FMT-CCR vs. FMT-Healthy		-2,22E-16	0		-2,22E-16	0,3422	3	3	9,177e-016	186
Ulceration										
Positive Control vs. FMT-CCR		0,3333	0,3333		0	0,3422	3	3	0	186
Positive Control vs. FMT-Healthy		0,3333	0,3333		0	0,3422	3	3	0	186
FMT-CCR vs. FMT-Healthy		0,3333	0,3333		0	0,3422	3	3	0	186
Pigment										
Positive Control vs. FMT-CCR		0	0		0	0,3422	3	3	0	186
Positive Control vs. FMT-Healthy		0	0		0	0,3422	3	3	0	186
FMT-CCR vs. FMT-Healthy		0	0		0	0,3422	3	3	0	186
Minerals										
Positive Control vs. FMT-CCR		0	0		0	0,3422	3	3	0	186
Positive Control vs. FMT-Healthy		0	0		0	0,3422	3	3	0	186
FMT-CCR vs. FMT-Healthy		0	0		0	0,3422	3	3	0	186
Necrosis										
Positive Control vs. FMT-CCR		0,3333	1		-0,6667	0,3422	3	3	2,755	186
Positive Control vs. FMT-Healthy		0,3333	-3,33E-16		0,3333	0,3422	3	3	1,378	186
FMT-CCR vs. FMT-Healthy		1	-3,33E-16		1	0,3422	3	3	4,133	186
Congestion										
Positive Control vs. FMT-CCR		0	0,6667		-0,6667	0,3422	3	3	2,755	186
Positive Control vs. FMT-Healthy		0	1		-1	0,3422	3	3	4,133	186
FMT-CCR vs. FMT-Healthy		0,6667	1		-0,3333	0,3422	3	3	1,378	186
Edema										
Positive Control vs. FMT-CCR	2,220e-016		-2,22E-16	4,441e-016		0,3422	3	3	1,835e-015	186
Positive Control vs. FMT-Healthy	2,220e-016		0,3333		-0,3333	0,3422	3	3	1,378	186
FMT-CCR vs. FMT-Healthy		-2,22E-16	0,3333		-0,3333	0,3422	3	3	1,378	186
Hemorrhage										
Positive Control vs. FMT-CCR		0	-3,33E-16	3,331e-016		0,3422	3	3	1,377e-015	186
Positive Control vs. FMT-Healthy		0	0		0	0,3422	3	3	0	186
FMT-CCR vs. FMT-Healthy		-3,33E-16	0		-3,33E-16	0,3422	3	3	1,377e-015	186
Thrombosis										
Positive Control vs. FMT-CCR		0	-3,33E-16	3,331e-016		0,3422	3	3	1,377e-015	186
Positive Control vs. FMT-Healthy		0	0		0	0,3422	3	3	0	186

FMT-CCR vs. FMT-Healthy	-3,33E-16	0	-3,33E-16	0,3422	3	3	1,377e-015	186
Fibrin								
Positive Control vs. FMT-CCR	0	-2,22E-16	2,220e-016	0,3422	3	3	9,177e-016	186
Positive Control vs. FMT-Healthy	0	0	0	0,3422	3	3	0	186
FMT-CCR vs. FMT-Healthy	-2,22E-16	0	-2,22E-16	0,3422	3	3	9,177e-016	186
Exocytosis								
Positive Control vs. FMT-CCR	0	-2,22E-16	2,220e-016	0,3422	3	3	9,177e-016	186
Positive Control vs. FMT-Healthy	0	0	0	0,3422	3	3	0	186
FMT-CCR vs. FMT-Healthy	-2,22E-16	0	-2,22E-16	0,3422	3	3	9,177e-016	186
Neutrophils								
Positive Control vs. FMT-CCR	0,6667	1	-0,3333	0,3422	3	3	1,378	186
Positive Control vs. FMT-Healthy	0,6667	1	-0,3333	0,3422	3	3	1,378	186
FMT-CCR vs. FMT-Healthy	1	1	0	0,3422	3	3	0	186
Eosinophils								
Positive Control vs. FMT-CCR	0	-3,33E-16	3,331e-016	0,3422	3	3	1,377e-015	186
Positive Control vs. FMT-Healthy	0	0	0	0,3422	3	3	0	186
FMT-CCR vs. FMT-Healthy	-3,33E-16	0	-3,33E-16	0,3422	3	3	1,377e-015	186
Macrophages								
Positive Control vs. FMT-CCR	1,333	1,667	-0,3333	0,3422	3	3	1,378	186
Positive Control vs. FMT-Healthy	1,333	1	0,3333	0,3422	3	3	1,378	186
FMT-CCR vs. FMT-Healthy	1,667	1	0,6667	0,3422	3	3	2,755	186
Lymphocytes								
Positive Control vs. FMT-CCR	1,667	1,667	0	0,3422	3	3	0	186
Positive Control vs. FMT-Healthy	1,667	1	0,6667	0,3422	3	3	2,755	186
FMT-CCR vs. FMT-Healthy	1,667	1	0,6667	0,3422	3	3	2,755	186
Plasma cells								
Positive Control vs. FMT-CCR	1,333	1,333	0	0,3422	3	3	0	186
Positive Control vs. FMT-Healthy	1,333	1	0,3333	0,3422	3	3	1,378	186
FMT-CCR vs. FMT-Healthy	1,333	1	0,3333	0,3422	3	3	1,378	186
Fibrosis								
Positive Control vs. FMT-CCR	2,333	1	1,333	0,3422	3	3	5,511	186
Positive Control vs. FMT-Healthy	2,333	1	1,333	0,3422	3	3	5,511	186
FMT-CCR vs. FMT-Healthy	1	1	-8,88E-16	0,3422	3	3	3,671e-015	186

Biological agents								
Positive Control vs. FMT-CCR	0	0	0	0,3422	3	3	0	186
Positive Control vs. FMT-Healthy	0	0	0	0,3422	3	3	0	186
FMT-CCR vs. FMT-Healthy	0	0	0	0,3422	3	3	0	186

Supplementary material

S4:

Table Analyzed	histopathogy of middle colonic tissue week 5				
Two-way ANOVA	Ordinary				
Alpha	0,05				
Source of Variation	% of total variation	P value	P value summary	Significant?	
Interaction	13,08	0,0005	***	Yes	
Row Factor	63,41	<0,0001	****	Yes	
Column Factor	2,463	<0,0001	****	Yes	
ANOVA table	SS	DF	MS	F (DFn, DFd)	P value
Interaction	17,39	60	0,2898	F (60, 186) = 1,925	P=0,0005
Row Factor	84,33	30	2,811	F (30, 186) = 18,67	P<0,0001
Column Factor	3,276	2	1,638	F (2, 186) = 10,88	P<0,0001
Residual	28	186	0,1505		

Within each row, compare columns (simple effects within rows)

Number of families	31
Number of comparisons per family	3
Alpha	0,05

Tukey's multiple comparisons test	Mean Diff,	95,00% CI of diff,	Significant?	Summary	Adjusted P Value
Epithelial atrophy					
Positive Control vs. FMT-CCR	0	-0,7485 to 0,7485	No	ns	>0,9999
Positive Control vs. FMT-Healthy	0	-0,7485 to 0,7485	No	ns	>0,9999
FMT-CCR vs. FMT-Healthy	0	-0,7485 to 0,7485	No	ns	>0,9999
Glandular atrophy					
Positive Control vs. FMT-CCR	-0,3333	-1,082 to 0,4151	No	ns	0,545
Positive Control vs. FMT-Healthy	-0,6667	-1,415 to 0,08180	No	ns	0,0917
FMT-CCR vs. FMT-Healthy	-0,3333	-1,082 to 0,4151	No	ns	0,545
Goblet cell					
Positive Control vs. FMT-CCR	0	-0,7485 to 0,7485	No	ns	>0,9999
Positive Control vs. FMT-Healthy	-0,3333	-1,082 to 0,4151	No	ns	0,545
FMT-CCR vs. FMT-Healthy	-0,3333	-1,082 to 0,4151	No	ns	0,545
Epithelial hyperplasia					

Positive Control vs. FMT-CCR	0,3333	-0,4151 to 1,082	No	ns	0,545
Positive Control vs. FMT-Healthy	0,3333	-0,4151 to 1,082	No	ns	0,545
FMT-CCR vs. FMT-Healthy	0	-0,7485 to 0,7485	No	ns	>0,9999
Glandular hyperplasia					
Positive Control vs. FMT-CCR	0,6667	-0,08180 to 1,415	No	ns	0,0917
Positive Control vs. FMT-Healthy	0,6667	-0,08180 to 1,415	No	ns	0,0917
FMT-CCR vs. FMT-Healthy	0	-0,7485 to 0,7485	No	ns	>0,9999
Mitosis					
Positive Control vs. FMT-CCR	-1,667	-2,415 to -0,9182	Yes	****	<0,0001
Positive Control vs. FMT-Healthy	0	-0,7485 to 0,7485	No	ns	>0,9999
FMT-CCR vs. FMT-Healthy	1,667	0,9182 to 2,415	Yes	****	<0,0001
Apoptosis					
Positive Control vs. FMT-CCR	0	-0,7485 to 0,7485	No	ns	>0,9999
Positive Control vs. FMT-Healthy	0	-0,7485 to 0,7485	No	ns	>0,9999
FMT-CCR vs. FMT-Healthy	0	-0,7485 to 0,7485	No	ns	>0,9999
Aberrant crypts					
Positive Control vs. FMT-CCR	0	-0,7485 to 0,7485	No	ns	>0,9999
Positive Control vs. FMT-Healthy	0	-0,7485 to 0,7485	No	ns	>0,9999
FMT-CCR vs. FMT-Healthy	0	-0,7485 to 0,7485	No	ns	>0,9999
Epithelial dysplasia					
Positive Control vs. FMT-CCR	-0,3333	-1,082 to 0,4151	No	ns	0,545
Positive Control vs. FMT-Healthy	-0,3333	-1,082 to 0,4151	No	ns	0,545
FMT-CCR vs. FMT-Healthy	0	-0,7485 to 0,7485	No	ns	>0,9999
Glandular dysplasia					
Positive Control vs. FMT-CCR	0	-0,7485 to 0,7485	No	ns	>0,9999
Positive Control vs. FMT-Healthy	0,3333	-0,4151 to 1,082	No	ns	0,545
FMT-CCR vs. FMT-Healthy	0,3333	-0,4151 to 1,082	No	ns	0,545
Neoplastic proliferation					
Positive Control vs. FMT-CCR	-1,333	-2,082 to -0,5849	Yes	***	0,0001
Positive Control vs. FMT-Healthy	0	-0,7485 to 0,7485	No	ns	>0,9999
FMT-CCR vs. FMT-Healthy	1,333	0,5849 to 2,082	Yes	***	0,0001
Neoplastic infiltration					
Positive Control vs. FMT-CCR	0	-0,7485 to 0,7485	No	ns	>0,9999
Positive Control vs. FMT-Healthy	0,6667	-0,08180 to 1,415	No	ns	0,0917
FMT-CCR vs. FMT-Healthy	0,6667	-0,08180 to 1,415	No	ns	0,0917

GALT hyperplasia					
Positive Control vs. FMT-CCR	-0,6667	-1,415 to 0,08180	No	ns	0,0917
Positive Control vs. FMT-Healthy	0,6667	-0,08180 to 1,415	No	ns	0,0917
FMT-CCR vs. FMT-Healthy	1,333	0,5849 to 2,082	Yes	***	0,0001
Erosion					
Positive Control vs. FMT-CCR	0,3333	-0,4151 to 1,082	No	ns	0,545
Positive Control vs. FMT-Healthy	0,3333	-0,4151 to 1,082	No	ns	0,545
FMT-CCR vs. FMT-Healthy	-2,22E-16	-0,7485 to 0,7485	No	ns	>0,9999
Ulceration					
Positive Control vs. FMT-CCR	0	-0,7485 to 0,7485	No	ns	>0,9999
Positive Control vs. FMT-Healthy	0	-0,7485 to 0,7485	No	ns	>0,9999
FMT-CCR vs. FMT-Healthy	0	-0,7485 to 0,7485	No	ns	>0,9999
Pigment					
Positive Control vs. FMT-CCR	0	-0,7485 to 0,7485	No	ns	>0,9999
Positive Control vs. FMT-Healthy	0	-0,7485 to 0,7485	No	ns	>0,9999
FMT-CCR vs. FMT-Healthy	0	-0,7485 to 0,7485	No	ns	>0,9999
Minerals					
Positive Control vs. FMT-CCR	0	-0,7485 to 0,7485	No	ns	>0,9999
Positive Control vs. FMT-Healthy	0	-0,7485 to 0,7485	No	ns	>0,9999
FMT-CCR vs. FMT-Healthy	0	-0,7485 to 0,7485	No	ns	>0,9999
Necrosis					
Positive Control vs. FMT-CCR	-0,3333	-1,082 to 0,4151	No	ns	0,545
Positive Control vs. FMT-Healthy	0	-0,7485 to 0,7485	No	ns	>0,9999
FMT-CCR vs. FMT-Healthy	0,3333	-0,4151 to 1,082	No	ns	0,545
Congestion					
Positive Control vs. FMT-CCR	-1	-1,748 to -0,2515	Yes	**	0,0053
Positive Control vs. FMT-Healthy	-0,6667	-1,415 to 0,08180	No	ns	0,0917
FMT-CCR vs. FMT-Healthy	0,3333	-0,4151 to 1,082	No	ns	0,545
Edema					
Positive Control vs. FMT-CCR	0	-0,7485 to 0,7485	No	ns	>0,9999
Positive Control vs. FMT-Healthy	0	-0,7485 to 0,7485	No	ns	>0,9999
FMT-CCR vs. FMT-Healthy	0	-0,7485 to 0,7485	No	ns	>0,9999
Hemorrhage					
Positive Control vs. FMT-CCR	2,220e-016	-0,7485 to 0,7485	No	ns	>0,9999
Positive Control vs. FMT-Healthy	0	-0,7485 to 0,7485	No	ns	>0,9999
FMT-CCR vs. FMT-Healthy	-2,22E-16	-0,7485 to 0,7485	No	ns	>0,9999

Thrombosis					
Positive Control vs. FMT-CCR	2,220e-016	-0,7485 to 0,7485	No	ns	>0,9999
Positive Control vs. FMT-Healthy	0	-0,7485 to 0,7485	No	ns	>0,9999
FMT-CCR vs. FMT-Healthy	-2,22E-16	-0,7485 to 0,7485	No	ns	>0,9999
Fibrin					
Positive Control vs. FMT-CCR	2,220e-016	-0,7485 to 0,7485	No	ns	>0,9999
Positive Control vs. FMT-Healthy	0	-0,7485 to 0,7485	No	ns	>0,9999
FMT-CCR vs. FMT-Healthy	-2,22E-16	-0,7485 to 0,7485	No	ns	>0,9999
Exocytosis					
Positive Control vs. FMT-CCR	2,220e-016	-0,7485 to 0,7485	No	ns	>0,9999
Positive Control vs. FMT-Healthy	2,220e-016	-0,7485 to 0,7485	No	ns	>0,9999
FMT-CCR vs. FMT-Healthy	0	-0,7485 to 0,7485	No	ns	>0,9999
Neutrophils					
Positive Control vs. FMT-CCR	0	-0,7485 to 0,7485	No	ns	>0,9999
Positive Control vs. FMT-Healthy	0	-0,7485 to 0,7485	No	ns	>0,9999
FMT-CCR vs. FMT-Healthy	0	-0,7485 to 0,7485	No	ns	>0,9999
Eosinophils					
Positive Control vs. FMT-CCR	2,220e-016	-0,7485 to 0,7485	No	ns	>0,9999
Positive Control vs. FMT-Healthy	0	-0,7485 to 0,7485	No	ns	>0,9999
FMT-CCR vs. FMT-Healthy	-2,22E-16	-0,7485 to 0,7485	No	ns	>0,9999
Macrophages					
Positive Control vs. FMT-CCR	0	-0,7485 to 0,7485	No	ns	>0,9999
Positive Control vs. FMT-Healthy	0,3333	-0,4151 to 1,082	No	ns	0,545
FMT-CCR vs. FMT-Healthy	0,3333	-0,4151 to 1,082	No	ns	0,545
Lymphocytes					
Positive Control vs. FMT-CCR	-0,6667	-1,415 to 0,08180	No	ns	0,0917
Positive Control vs. FMT-Healthy	0,3333	-0,4151 to 1,082	No	ns	0,545
FMT-CCR vs. FMT-Healthy	1	0,2515 to 1,748	Yes	**	0,0053
Plasma cells					
Positive Control vs. FMT-CCR	-0,6667	-1,415 to 0,08180	No	ns	0,0917
Positive Control vs. FMT-Healthy	0	-0,7485 to 0,7485	No	ns	>0,9999
FMT-CCR vs. FMT-Healthy	0,6667	-0,08180 to 1,415	No	ns	0,0917
Fibrosis					
Positive Control vs. FMT-CCR	0	-0,7485 to 0,7485	No	ns	>0,9999
Positive Control vs. FMT-Healthy	0,6667	-0,08180 to 1,415	No	ns	0,0917

FMT-CCR vs. FMT-Healthy	0,6667	-0,08180 to 1,415	No	ns	0,0917
Biological agents					
Positive Control vs. FMT-CCR	0	-0,7485 to 0,7485	No	ns	>0,9999
Positive Control vs. FMT-Healthy	0	-0,7485 to 0,7485	No	ns	>0,9999
FMT-CCR vs. FMT-Healthy	0	-0,7485 to 0,7485	No	ns	>0,9999

Test details	Mean 1	Mean 2	Mean Diff,	SE of diff,	N1	N2	q	DF
Epithelial atrophy								
Positive Control vs. FMT-CCR	0	0	0	0,3168	3	3	0	186
Positive Control vs. FMT-Healthy	0	0	0	0,3168	3	3	0	186
FMT-CCR vs. FMT-Healthy	0	0	0	0,3168	3	3	0	186
Glandular atrophy								
Positive Control vs. FMT-CCR	0	0,3333	-0,3333	0,3168	3	3	1,488	186
Positive Control vs. FMT-Healthy	0	0,6667	-0,6667	0,3168	3	3	2,976	186
FMT-CCR vs. FMT-Healthy	0,3333	0,6667	-0,3333	0,3168	3	3	1,488	186
Goblet cell								
Positive Control vs. FMT-CCR	0	0	0	0,3168	3	3	0	186
Positive Control vs. FMT-Healthy	0	0,3333	-0,3333	0,3168	3	3	1,488	186
FMT-CCR vs. FMT-Healthy	0	0,3333	-0,3333	0,3168	3	3	1,488	186
Epithelial hyperplasia								
Positive Control vs. FMT-CCR	0,6667	0,3333	0,3333	0,3168	3	3	1,488	186
Positive Control vs. FMT-Healthy	0,6667	0,3333	0,3333	0,3168	3	3	1,488	186
FMT-CCR vs. FMT-Healthy	0,3333	0,3333	0	0,3168	3	3	0	186
Glandular hyperplasia								
Positive Control vs. FMT-CCR	1,333	0,6667	0,6667	0,3168	3	3	2,976	186
Positive Control vs. FMT-Healthy	1,333	0,6667	0,6667	0,3168	3	3	2,976	186
FMT-CCR vs. FMT-Healthy	0,6667	0,6667	0	0,3168	3	3	0	186
Mitosis								
Positive Control vs. FMT-CCR	1	2,667	-1,667	0,3168	3	3	7,44	186
Positive Control vs. FMT-Healthy	1	1	0	0,3168	3	3	0	186
FMT-CCR vs. FMT-Healthy	2,667	1	1,667	0,3168	3	3	7,44	186
Apoptosis								
Positive Control vs. FMT-CCR	0	0	0	0,3168	3	3	0	186
Positive Control vs. FMT-Healthy	0	0	0	0,3168	3	3	0	186
FMT-CCR vs. FMT-Healthy	0	0	0	0,3168	3	3	0	186

Aberrant crypts								
Positive Control vs. FMT-CCR	1	1	0	0,3168	3	3	0	186
Positive Control vs. FMT-Healthy	1	1	0	0,3168	3	3	0	186
FMT-CCR vs. FMT-Healthy	1	1	0	0,3168	3	3	0	186
Epithelial dysplasia								
Positive Control vs. FMT-CCR	0	0,3333	-0,3333	0,3168	3	3	1,488	186
Positive Control vs. FMT-Healthy	0	0,3333	-0,3333	0,3168	3	3	1,488	186
FMT-CCR vs. FMT-Healthy	0,3333	0,3333	0	0,3168	3	3	0	186
Glandular dysplasia								
Positive Control vs. FMT-CCR	1	1	0	0,3168	3	3	0	186
Positive Control vs. FMT-Healthy	1	0,6667	0,3333	0,3168	3	3	1,488	186
FMT-CCR vs. FMT-Healthy	1	0,6667	0,3333	0,3168	3	3	1,488	186
Neoplastic proliferation								
Positive Control vs. FMT-CCR	0,3333	1,667	-1,333	0,3168	3	3	5,952	186
Positive Control vs. FMT-Healthy	0,3333	0,3333	0	0,3168	3	3	0	186
FMT-CCR vs. FMT-Healthy	1,667	0,3333	1,333	0,3168	3	3	5,952	186
Neoplastic infiltration								
Positive Control vs. FMT-CCR	1	1	0	0,3168	3	3	0	186
Positive Control vs. FMT-Healthy	1	0,3333	0,6667	0,3168	3	3	2,976	186
FMT-CCR vs. FMT-Healthy	1	0,3333	0,6667	0,3168	3	3	2,976	186
GALT hyperplasia								
Positive Control vs. FMT-CCR	1,333	2	-0,6667	0,3168	3	3	2,976	186
Positive Control vs. FMT-Healthy	1,333	0,6667	0,6667	0,3168	3	3	2,976	186
FMT-CCR vs. FMT-Healthy	2	0,6667	1,333	0,3168	3	3	5,952	186
Erosion								
Positive Control vs. FMT-CCR	0,3333	-2,22E-16	0,3333	0,3168	3	3	1,488	186
Positive Control vs. FMT-Healthy	0,3333	0	0,3333	0,3168	3	3	1,488	186
FMT-CCR vs. FMT-Healthy	-2,22E-16	0	-2,22E-16	0,3168	3	3	9,912e-016	186
Ulceration								
Positive Control vs. FMT-CCR	0,6667	0,6667	0	0,3168	3	3	0	186
Positive Control vs. FMT-Healthy	0,6667	0,6667	0	0,3168	3	3	0	186
FMT-CCR vs. FMT-Healthy	0,6667	0,6667	0	0,3168	3	3	0	186
Pigment								
Positive Control vs. FMT-CCR	0	0	0	0,3168	3	3	0	186
Positive Control vs. FMT-Healthy	0	0	0	0,3168	3	3	0	186

FMT-CCR vs. FMT-Healthy	0	0	0	0,3168	3	3	0	186
Minerals								
Positive Control vs. FMT-CCR	0	0	0	0,3168	3	3	0	186
Positive Control vs. FMT-Healthy	0	0	0	0,3168	3	3	0	186
FMT-CCR vs. FMT-Healthy	0	0	0	0,3168	3	3	0	186
Necrosis								
Positive Control vs. FMT-CCR	0,6667	1	-0,3333	0,3168	3	3	1,488	186
Positive Control vs. FMT-Healthy	0,6667	0,6667	0	0,3168	3	3	0	186
FMT-CCR vs. FMT-Healthy	1	0,6667	0,3333	0,3168	3	3	1,488	186
Congestion								
Positive Control vs. FMT-CCR	0	1	-1	0,3168	3	3	4,464	186
Positive Control vs. FMT-Healthy	0	0,6667	-0,6667	0,3168	3	3	2,976	186
FMT-CCR vs. FMT-Healthy	1	0,6667	0,3333	0,3168	3	3	1,488	186
Edema								
Positive Control vs. FMT-CCR	0	0	0	0,3168	3	3	0	186
Positive Control vs. FMT-Healthy	0	0	0	0,3168	3	3	0	186
FMT-CCR vs. FMT-Healthy	0	0	0	0,3168	3	3	0	186
Hemorrhage								
Positive Control vs. FMT-CCR	0	-2,22E-16	2,220e-016	0,3168	3	3	9,912e-016	186
Positive Control vs. FMT-Healthy	0	0	0	0,3168	3	3	0	186
FMT-CCR vs. FMT-Healthy	-2,22E-16	0	-2,22E-16	0,3168	3	3	9,912e-016	186
Thrombosis								
Positive Control vs. FMT-CCR	0	-2,22E-16	2,220e-016	0,3168	3	3	9,912e-016	186
Positive Control vs. FMT-Healthy	0	0	0	0,3168	3	3	0	186
FMT-CCR vs. FMT-Healthy	-2,22E-16	0	-2,22E-16	0,3168	3	3	9,912e-016	186
Fibrin								
Positive Control vs. FMT-CCR	0	-2,22E-16	2,220e-016	0,3168	3	3	9,912e-016	186
Positive Control vs. FMT-Healthy	0	0	0	0,3168	3	3	0	186
FMT-CCR vs. FMT-Healthy	-2,22E-16	0	-2,22E-16	0,3168	3	3	9,912e-016	186
Exocytosis								
Positive Control vs. FMT-CCR	0	-2,22E-16	2,220e-016	0,3168	3	3	9,912e-016	186
Positive Control vs. FMT-Healthy	0	-2,22E-16	2,220e-016	0,3168	3	3	9,912e-016	186
FMT-CCR vs. FMT-Healthy	-2,22E-16	-2,22E-16	0	0,3168	3	3	0	186
Neutrophils								
Positive Control vs. FMT-CCR	1	1	0	0,3168	3	3	0	186

Positive Control vs. FMT-Healthy	1	1	0	0,3168	3	3	0	186
FMT-CCR vs. FMT-Healthy	1	1	0	0,3168	3	3	0	186
Eosinophils								
Positive Control vs. FMT-CCR	0	-2,22E-16	2,220e-016	0,3168	3	3	9,912e-016	186
Positive Control vs. FMT-Healthy	0	0	0	0,3168	3	3	0	186
FMT-CCR vs. FMT-Healthy	-2,22E-16	0	-2,22E-16	0,3168	3	3	9,912e-016	186
Macrophages								
Positive Control vs. FMT-CCR	1,667	1,667	0	0,3168	3	3	0	186
Positive Control vs. FMT-Healthy	1,667	1,333	0,3333	0,3168	3	3	1,488	186
FMT-CCR vs. FMT-Healthy	1,667	1,333	0,3333	0,3168	3	3	1,488	186
Lymphocytes								
Positive Control vs. FMT-CCR	1,333	2	-0,6667	0,3168	3	3	2,976	186
Positive Control vs. FMT-Healthy	1,333	1	0,3333	0,3168	3	3	1,488	186
FMT-CCR vs. FMT-Healthy	2	1	1	0,3168	3	3	4,464	186
Plasma cells								
Positive Control vs. FMT-CCR	1	1,667	-0,6667	0,3168	3	3	2,976	186
Positive Control vs. FMT-Healthy	1	1	0	0,3168	3	3	0	186
FMT-CCR vs. FMT-Healthy	1,667	1	0,6667	0,3168	3	3	2,976	186
Fibrosis								
Positive Control vs. FMT-CCR	1,667	1,667	0	0,3168	3	3	0	186
Positive Control vs. FMT-Healthy	1,667	1	0,6667	0,3168	3	3	2,976	186
FMT-CCR vs. FMT-Healthy	1,667	1	0,6667	0,3168	3	3	2,976	186
Biological agents								
Positive Control vs. FMT-CCR	0	0	0	0,3168	3	3	0	186
Positive Control vs. FMT-Healthy	0	0	0	0,3168	3	3	0	186
FMT-CCR vs. FMT-Healthy	0	0	0	0,3168	3	3	0	186

Supplementary material

S5:

Table Analyzed	Histopathology of Middle Colonic Tissue Week 12				
Two-way ANOVA	Ordinary				
Alpha	0,05				
Source of Variation	% of total variation	P value	P value summary	Significant?	
Interaction	7,154	0,0411	*	Yes	
Row Factor	76,34	<0,0001	****	Yes	
Column Factor	0,8473	0,0074	**	Yes	
ANOVA table	SS	DF	MS	F (DFn, DFd)	P value
Interaction	9,14	60	0,1523	F (60, 186) = 1,417	P=0,0411
Row Factor	97,53	30	3,251	F (30, 186) = 30,23	P<0,0001
Column Factor	1,082	2	0,5412	F (2, 186) = 5,033	P=0,0074
Residual	20	186	0,1075		

Within each row, compare columns (simple effects within rows)

Number of families	31
Number of comparisons per family	3
Alpha	0,05

Tukey's multiple comparisons test	Mean Diff,	95,00% CI of diff,	Significant?	Summary	Adjusted P Value
Epithelial atrophy					
Positive Control vs. FMT-CCR	2,220e-016	-0,6326 to 0,6326	No	ns	>0,9999
Positive Control vs. FMT-Healthy	0	-0,6326 to 0,6326	No	ns	>0,9999
FMT-CCR vs. FMT-Healthy	-2,22E-16	-0,6326 to 0,6326	No	ns	>0,9999
Glandular atrophy					
Positive Control vs. FMT-CCR	-0,3333	-0,9659 to 0,2992	No	ns	0,4283
Positive Control vs. FMT-Healthy	0	-0,6326 to 0,6326	No	ns	>0,9999
FMT-CCR vs. FMT-Healthy	0,3333	-0,2992 to 0,9659	No	ns	0,4283
Goblet cell					
Positive Control vs. FMT-CCR	0	-0,6326 to 0,6326	No	ns	>0,9999
Positive Control vs. FMT-Healthy	0,3333	-0,2992 to 0,9659	No	ns	0,4283
FMT-CCR vs. FMT-Healthy	0,3333	-0,2992 to 0,9659	No	ns	0,4283
Epithelial hyperplasia					
Positive Control vs. FMT-CCR	0	-0,6326 to 0,6326	No	ns	>0,9999

Positive Control vs. FMT-Healthy	0	-0,6326 to 0,6326	No	ns	>0,9999
FMT-CCR vs. FMT-Healthy	0	-0,6326 to 0,6326	No	ns	>0,9999
Glandular hyperplasia					
Positive Control vs. FMT-CCR	0	-0,6326 to 0,6326	No	ns	>0,9999
Positive Control vs. FMT-Healthy	-0,3333	-0,9659 to 0,2992	No	ns	0,4283
FMT-CCR vs. FMT-Healthy	-0,3333	-0,9659 to 0,2992	No	ns	0,4283
Mitosis					
Positive Control vs. FMT-CCR	-0,6667	-1,299 to -0,03410	Yes	*	0,0362
Positive Control vs. FMT-Healthy	-1	-1,633 to -0,3674	Yes	***	0,0007
FMT-CCR vs. FMT-Healthy	-0,3333	-0,9659 to 0,2992	No	ns	0,4283
Apoptosis					
Positive Control vs. FMT-CCR	0,6667	0,03410 to 1,299	Yes	*	0,0362
Positive Control vs. FMT-Healthy	0,3333	-0,2992 to 0,9659	No	ns	0,4283
FMT-CCR vs. FMT-Healthy	-0,3333	-0,9659 to 0,2992	No	ns	0,4283
Aberrant crypts					
Positive Control vs. FMT-CCR	-0,3333	-0,9659 to 0,2992	No	ns	0,4283
Positive Control vs. FMT-Healthy	-0,3333	-0,9659 to 0,2992	No	ns	0,4283
FMT-CCR vs. FMT-Healthy	0	-0,6326 to 0,6326	No	ns	>0,9999
Epithelial dysplasia					
Positive Control vs. FMT-CCR	0	-0,6326 to 0,6326	No	ns	>0,9999
Positive Control vs. FMT-Healthy	0	-0,6326 to 0,6326	No	ns	>0,9999
FMT-CCR vs. FMT-Healthy	0	-0,6326 to 0,6326	No	ns	>0,9999
Glandular dysplasia					
Positive Control vs. FMT-CCR	-0,3333	-0,9659 to 0,2992	No	ns	0,4283
Positive Control vs. FMT-Healthy	-0,3333	-0,9659 to 0,2992	No	ns	0,4283
FMT-CCR vs. FMT-Healthy	0	-0,6326 to 0,6326	No	ns	>0,9999
Neoplastic proliferation					
Positive Control vs. FMT-CCR	-1	-1,633 to -0,3674	Yes	***	0,0007
Positive Control vs. FMT-Healthy	0	-0,6326 to 0,6326	No	ns	>0,9999
FMT-CCR vs. FMT-Healthy	1	0,3674 to 1,633	Yes	***	0,0007
Neoplastic infiltration					
Positive Control vs. FMT-CCR	0	-0,6326 to 0,6326	No	ns	>0,9999
Positive Control vs. FMT-Healthy	0	-0,6326 to 0,6326	No	ns	>0,9999
FMT-CCR vs. FMT-Healthy	0	-0,6326 to 0,6326	No	ns	>0,9999
GALT hyperplasia					

Positive Control vs. FMT-CCR		-1	-1,633 to -0,3674	Yes	***	0,0007
Positive Control vs. FMT-Healthy		0	-0,6326 to 0,6326	No	ns	>0,9999
FMT-CCR vs. FMT-Healthy		1	0,3674 to 1,633	Yes	***	0,0007
Erosion						
Positive Control vs. FMT-CCR		0,3333	-0,2992 to 0,9659	No	ns	0,4283
Positive Control vs. FMT-Healthy		0,3333	-0,2992 to 0,9659	No	ns	0,4283
FMT-CCR vs. FMT-Healthy		-2,22E-16	-0,6326 to 0,6326	No	ns	>0,9999
Ulceration						
Positive Control vs. FMT-CCR		0	-0,6326 to 0,6326	No	ns	>0,9999
Positive Control vs. FMT-Healthy		0	-0,6326 to 0,6326	No	ns	>0,9999
FMT-CCR vs. FMT-Healthy		0	-0,6326 to 0,6326	No	ns	>0,9999
Pigment						
Positive Control vs. FMT-CCR		0	-0,6326 to 0,6326	No	ns	>0,9999
Positive Control vs. FMT-Healthy		0	-0,6326 to 0,6326	No	ns	>0,9999
FMT-CCR vs. FMT-Healthy		0	-0,6326 to 0,6326	No	ns	>0,9999
Minerals						
Positive Control vs. FMT-CCR		0	-0,6326 to 0,6326	No	ns	>0,9999
Positive Control vs. FMT-Healthy		0	-0,6326 to 0,6326	No	ns	>0,9999
FMT-CCR vs. FMT-Healthy		0	-0,6326 to 0,6326	No	ns	>0,9999
Necrosis						
Positive Control vs. FMT-CCR		-0,3333	-0,9659 to 0,2992	No	ns	0,4283
Positive Control vs. FMT-Healthy		-0,3333	-0,9659 to 0,2992	No	ns	0,4283
FMT-CCR vs. FMT-Healthy		0	-0,6326 to 0,6326	No	ns	>0,9999
Congestion						
Positive Control vs. FMT-CCR		-1	-1,633 to -0,3674	Yes	***	0,0007
Positive Control vs. FMT-Healthy		-0,3333	-0,9659 to 0,2992	No	ns	0,4283
FMT-CCR vs. FMT-Healthy		0,6667	0,03410 to 1,299	Yes	*	0,0362
Edema						
Positive Control vs. FMT-CCR	2,220e-016		-0,6326 to 0,6326	No	ns	>0,9999
Positive Control vs. FMT-Healthy		0	-0,6326 to 0,6326	No	ns	>0,9999
FMT-CCR vs. FMT-Healthy		-2,22E-16	-0,6326 to 0,6326	No	ns	>0,9999
Hemorrhage						
Positive Control vs. FMT-CCR	2,220e-016		-0,6326 to 0,6326	No	ns	>0,9999
Positive Control vs. FMT-Healthy		0	-0,6326 to 0,6326	No	ns	>0,9999
FMT-CCR vs. FMT-Healthy		-2,22E-16	-0,6326 to 0,6326	No	ns	>0,9999

Thrombosis						
Positive Control vs. FMT-CCR	2,220e-016		-0,6326 to 0,6326	No	ns	>0,9999
Positive Control vs. FMT-Healthy		0	-0,6326 to 0,6326	No	ns	>0,9999
FMT-CCR vs. FMT-Healthy		-2,22E-16	-0,6326 to 0,6326	No	ns	>0,9999
Fibrin						
Positive Control vs. FMT-CCR	2,220e-016		-0,6326 to 0,6326	No	ns	>0,9999
Positive Control vs. FMT-Healthy		0	-0,6326 to 0,6326	No	ns	>0,9999
FMT-CCR vs. FMT-Healthy		-2,22E-16	-0,6326 to 0,6326	No	ns	>0,9999
Exocytosis						
Positive Control vs. FMT-CCR	3,331e-016		-0,6326 to 0,6326	No	ns	>0,9999
Positive Control vs. FMT-Healthy		0	-0,6326 to 0,6326	No	ns	>0,9999
FMT-CCR vs. FMT-Healthy		-3,33E-16	-0,6326 to 0,6326	No	ns	>0,9999
Neutrophils						
Positive Control vs. FMT-CCR		0	-0,6326 to 0,6326	No	ns	>0,9999
Positive Control vs. FMT-Healthy		0	-0,6326 to 0,6326	No	ns	>0,9999
FMT-CCR vs. FMT-Healthy		0	-0,6326 to 0,6326	No	ns	>0,9999
Eosinophils						
Positive Control vs. FMT-CCR	3,331e-016		-0,6326 to 0,6326	No	ns	>0,9999
Positive Control vs. FMT-Healthy		0	-0,6326 to 0,6326	No	ns	>0,9999
FMT-CCR vs. FMT-Healthy		-3,33E-16	-0,6326 to 0,6326	No	ns	>0,9999
Macrophages						
Positive Control vs. FMT-CCR		0	-0,6326 to 0,6326	No	ns	>0,9999
Positive Control vs. FMT-Healthy		0	-0,6326 to 0,6326	No	ns	>0,9999
FMT-CCR vs. FMT-Healthy		0	-0,6326 to 0,6326	No	ns	>0,9999
Lymphocytes						
Positive Control vs. FMT-CCR		0	-0,6326 to 0,6326	No	ns	>0,9999
Positive Control vs. FMT-Healthy		0	-0,6326 to 0,6326	No	ns	>0,9999
FMT-CCR vs. FMT-Healthy		0	-0,6326 to 0,6326	No	ns	>0,9999
Plasma cells						
Positive Control vs. FMT-CCR		-0,6667	-1,299 to -0,03410	Yes	*	0,0362
Positive Control vs. FMT-Healthy		0	-0,6326 to 0,6326	No	ns	>0,9999
FMT-CCR vs. FMT-Healthy		0,6667	0,03410 to 1,299	Yes	*	0,0362
Fibrosis						
Positive Control vs. FMT-CCR		0	-0,6326 to 0,6326	No	ns	>0,9999
Positive Control vs. FMT-Healthy		0	-0,6326 to 0,6326	No	ns	>0,9999
FMT-CCR vs. FMT-Healthy		0	-0,6326 to 0,6326	No	ns	>0,9999

Biological agents					
Positive Control vs. FMT-CCR	0	-0,6326 to 0,6326	No	ns	>0,9999
Positive Control vs. FMT-Healthy	0	-0,6326 to 0,6326	No	ns	>0,9999
FMT-CCR vs. FMT-Healthy	0	-0,6326 to 0,6326	No	ns	>0,9999

Test details	Mean 1	Mean 2	Mean Diff,	SE of diff,	N1	N2	q	DF
Epithelial atrophy								
Positive Control vs. FMT-CCR	0	-2,22E-16	2,220e-016	0,2677	3	3	1,173e-015	186
Positive Control vs. FMT-Healthy	0	0	0	0,2677	3	3	0	186
FMT-CCR vs. FMT-Healthy	-2,22E-16	0	-2,22E-16	0,2677	3	3	1,173e-015	186
Glandular atrophy								
Positive Control vs. FMT-CCR	0	0,3333	-0,3333	0,2677	3	3	1,761	186
Positive Control vs. FMT-Healthy	0	0	0	0,2677	3	3	0	186
FMT-CCR vs. FMT-Healthy	0,3333	0	0,3333	0,2677	3	3	1,761	186
Goblet cell								
Positive Control vs. FMT-CCR	0,3333	0,3333	0	0,2677	3	3	0	186
Positive Control vs. FMT-Healthy	0,3333	0	0,3333	0,2677	3	3	1,761	186
FMT-CCR vs. FMT-Healthy	0,3333	0	0,3333	0,2677	3	3	1,761	186
Epithelial hyperplasia								
Positive Control vs. FMT-CCR	0	0	0	0,2677	3	3	0	186
Positive Control vs. FMT-Healthy	0	0	0	0,2677	3	3	0	186
FMT-CCR vs. FMT-Healthy	0	0	0	0,2677	3	3	0	186
Glandular hyperplasia								
Positive Control vs. FMT-CCR	0,6667	0,6667	0	0,2677	3	3	0	186
Positive Control vs. FMT-Healthy	0,6667	1	-0,3333	0,2677	3	3	1,761	186
FMT-CCR vs. FMT-Healthy	0,6667	1	-0,3333	0,2677	3	3	1,761	186
Mitosis								
Positive Control vs. FMT-CCR	1	1,667	-0,6667	0,2677	3	3	3,521	186
Positive Control vs. FMT-Healthy	1	2	-1	0,2677	3	3	5,282	186
FMT-CCR vs. FMT-Healthy	1,667	2	-0,3333	0,2677	3	3	1,761	186
Apoptosis								
Positive Control vs. FMT-CCR	0,6667	0	0,6667	0,2677	3	3	3,521	186
Positive Control vs. FMT-Healthy	0,6667	0,3333	0,3333	0,2677	3	3	1,761	186
FMT-CCR vs. FMT-Healthy	0	0,3333	-0,3333	0,2677	3	3	1,761	186

Aberrant crypts								
Positive Control vs. FMT-CCR	0,3333	0,6667	-0,3333	0,2677	3	3	1,761	186
Positive Control vs. FMT-Healthy	0,3333	0,6667	-0,3333	0,2677	3	3	1,761	186
FMT-CCR vs. FMT-Healthy	0,6667	0,6667	0	0,2677	3	3	0	186
Epithelial dysplasia								
Positive Control vs. FMT-CCR	0	0	0	0,2677	3	3	0	186
Positive Control vs. FMT-Healthy	0	0	0	0,2677	3	3	0	186
FMT-CCR vs. FMT-Healthy	0	0	0	0,2677	3	3	0	186
Glandular dysplasia								
Positive Control vs. FMT-CCR	0,3333	0,6667	-0,3333	0,2677	3	3	1,761	186
Positive Control vs. FMT-Healthy	0,3333	0,6667	-0,3333	0,2677	3	3	1,761	186
FMT-CCR vs. FMT-Healthy	0,6667	0,6667	0	0,2677	3	3	0	186
Neoplastic proliferation								
Positive Control vs. FMT-CCR	2	3	-1	0,2677	3	3	5,282	186
Positive Control vs. FMT-Healthy	2	2	0	0,2677	3	3	0	186
FMT-CCR vs. FMT-Healthy	3	2	1	0,2677	3	3	5,282	186
Neoplastic infiltration								
Positive Control vs. FMT-CCR	1	1	0	0,2677	3	3	0	186
Positive Control vs. FMT-Healthy	1	1	0	0,2677	3	3	0	186
FMT-CCR vs. FMT-Healthy	1	1	0	0,2677	3	3	0	186
GALT hyperplasia								
Positive Control vs. FMT-CCR	1	2	-1	0,2677	3	3	5,282	186
Positive Control vs. FMT-Healthy	1	1	0	0,2677	3	3	0	186
FMT-CCR vs. FMT-Healthy	2	1	1	0,2677	3	3	5,282	186
Erosion								
Positive Control vs. FMT-CCR	0,3333	-2,22E-16	0,3333	0,2677	3	3	1,761	186
Positive Control vs. FMT-Healthy	0,3333	0	0,3333	0,2677	3	3	1,761	186
FMT-CCR vs. FMT-Healthy	-2,22E-16	0	-2,22E-16	0,2677	3	3	1,173e-015	186
Ulceration								
Positive Control vs. FMT-CCR	0	0	0	0,2677	3	3	0	186
Positive Control vs. FMT-Healthy	0	0	0	0,2677	3	3	0	186
FMT-CCR vs. FMT-Healthy	0	0	0	0,2677	3	3	0	186
Pigment								
Positive Control vs. FMT-CCR	0	0	0	0,2677	3	3	0	186
Positive Control vs. FMT-Healthy	0	0	0	0,2677	3	3	0	186
FMT-CCR vs. FMT-Healthy	0	0	0	0,2677	3	3	0	186

Minerals								
Positive Control vs. FMT-CCR	0	0	0	0,2677	3	3	0	186
Positive Control vs. FMT-Healthy	0	0	0	0,2677	3	3	0	186
FMT-CCR vs. FMT-Healthy	0	0	0	0,2677	3	3	0	186
Necrosis								
Positive Control vs. FMT-CCR	0,6667	1	-0,3333	0,2677	3	3	1,761	186
Positive Control vs. FMT-Healthy	0,6667	1	-0,3333	0,2677	3	3	1,761	186
FMT-CCR vs. FMT-Healthy	1	1	0	0,2677	3	3	0	186
Congestion								
Positive Control vs. FMT-CCR	-3,33E-16	1	-1	0,2677	3	3	5,282	186
Positive Control vs. FMT-Healthy	-3,33E-16	0,3333	-0,3333	0,2677	3	3	1,761	186
FMT-CCR vs. FMT-Healthy	1	0,3333	0,6667	0,2677	3	3	3,521	186
Edema								
Positive Control vs. FMT-CCR	0	-2,22E-16	2,220e-016	0,2677	3	3	1,173e-015	186
Positive Control vs. FMT-Healthy	0	0	0	0,2677	3	3	0	186
FMT-CCR vs. FMT-Healthy	-2,22E-16	0	-2,22E-16	0,2677	3	3	1,173e-015	186
Hemorrhage								
Positive Control vs. FMT-CCR	0	-2,22E-16	2,220e-016	0,2677	3	3	1,173e-015	186
Positive Control vs. FMT-Healthy	0	0	0	0,2677	3	3	0	186
FMT-CCR vs. FMT-Healthy	-2,22E-16	0	-2,22E-16	0,2677	3	3	1,173e-015	186
Thrombosis								
Positive Control vs. FMT-CCR	0	-2,22E-16	2,220e-016	0,2677	3	3	1,173e-015	186
Positive Control vs. FMT-Healthy	0	0	0	0,2677	3	3	0	186
FMT-CCR vs. FMT-Healthy	-2,22E-16	0	-2,22E-16	0,2677	3	3	1,173e-015	186
Fibrin								
Positive Control vs. FMT-CCR	0	-2,22E-16	2,220e-016	0,2677	3	3	1,173e-015	186
Positive Control vs. FMT-Healthy	0	0	0	0,2677	3	3	0	186
FMT-CCR vs. FMT-Healthy	-2,22E-16	0	-2,22E-16	0,2677	3	3	1,173e-015	186
Exocytosis								
Positive Control vs. FMT-CCR	0	-3,33E-16	3,331e-016	0,2677	3	3	1,759e-015	186
Positive Control vs. FMT-Healthy	0	0	0	0,2677	3	3	0	186
FMT-CCR vs. FMT-Healthy	-3,33E-16	0	-3,33E-16	0,2677	3	3	1,759e-015	186
Neutrophils								
Positive Control vs. FMT-CCR	1	1	0	0,2677	3	3	0	186
Positive Control vs. FMT-Healthy	1	1	0	0,2677	3	3	0	186

FMT-CCR vs. FMT-Healthy	1	1	0	0,2677	3	3	0	186
Eosinophils								
Positive Control vs. FMT-CCR	0	-3,33E-16	3,331e-016	0,2677	3	3	1,759e-015	186
Positive Control vs. FMT-Healthy	0	0	0	0,2677	3	3	0	186
FMT-CCR vs. FMT-Healthy	-3,33E-16	0	-3,33E-16	0,2677	3	3	1,759e-015	186
Macrophages								
Positive Control vs. FMT-CCR	1	1	0	0,2677	3	3	0	186
Positive Control vs. FMT-Healthy	1	1	0	0,2677	3	3	0	186
FMT-CCR vs. FMT-Healthy	1	1	0	0,2677	3	3	0	186
Lymphocytes								
Positive Control vs. FMT-CCR	1	1	0	0,2677	3	3	0	186
Positive Control vs. FMT-Healthy	1	1	0	0,2677	3	3	0	186
FMT-CCR vs. FMT-Healthy	1	1	0	0,2677	3	3	0	186
Plasma cells								
Positive Control vs. FMT-CCR	1	1,667	-0,6667	0,2677	3	3	3,521	186
Positive Control vs. FMT-Healthy	1	1	0	0,2677	3	3	0	186
FMT-CCR vs. FMT-Healthy	1,667	1	0,6667	0,2677	3	3	3,521	186
Fibrosis								
Positive Control vs. FMT-CCR	1	1	0	0,2677	3	3	0	186
Positive Control vs. FMT-Healthy	1	1	0	0,2677	3	3	0	186
FMT-CCR vs. FMT-Healthy	1	1	0	0,2677	3	3	0	186
Biological agents								
Positive Control vs. FMT-CCR	0	0	0	0,2677	3	3	0	186
Positive Control vs. FMT-Healthy	0	0	0	0,2677	3	3	0	186
FMT-CCR vs. FMT-Healthy	0	0	0	0,2677	3	3	0	186

Supplementary material

S6:

Table Analyzed	Histopathology of distal Colonic Tissue Week 12
Two-way ANOVA	Ordinary
Alpha	0,05

Source of Variation	% of total variation	P value	P value summary	Significant?
Interaction	10,05	0,0009	***	Yes
Row Factor	72,41	<0,0001	****	Yes
Column Factor	0,8142	0,012	*	Yes

ANOVA table	SS	DF	MS	F (DFn, DFd)	P value
Interaction	15,21	60	0,2535	F (60, 186) = 1,86	P=0,0009
Row Factor	109,6	30	3,655	F (30, 186) = 26,8	P<0,0001
Column Factor	1,233	2	0,6165	F (2, 186) = 4,526	P=0,0120
Residual	25,33	186	0,1362		

Within each row, compare columns (simple effects within rows)

Number of families	31
Number of comparisons per family	3
Alpha	0,05

Tukey's multiple comparisons test	Mean Diff,	95,00% CI of diff,	Significant?	Summary	Adjusted P Value
Epithelial atrophy					
Positive Control vs. FMT-CCR	0	-0,7119 to 0,7119	No	ns	>0,9999
Positive Control vs. FMT-Healthy	-2,22E-16	-0,7119 to 0,7119	No	ns	>0,9999
FMT-CCR vs. FMT-Healthy	-2,22E-16	-0,7119 to 0,7119	No	ns	>0,9999
Glandular atrophy					
Positive Control vs. FMT-CCR	0	-0,7119 to 0,7119	No	ns	>0,9999
Positive Control vs. FMT-Healthy	0	-0,7119 to 0,7119	No	ns	>0,9999
FMT-CCR vs. FMT-Healthy	0	-0,7119 to 0,7119	No	ns	>0,9999
Goblet cell					
Positive Control vs. FMT-CCR	0,6667	-0,04527 to 1,379	No	ns	0,0717
Positive Control vs. FMT-Healthy	0,3333	-0,3786 to 1,045	No	ns	0,5114
FMT-CCR vs. FMT-Healthy	-0,3333	-1,045 to 0,3786	No	ns	0,5114
Epithelial hyperplasia					
Positive Control vs. FMT-CCR	0	-0,7119 to 0,7119	No	ns	>0,9999

Positive Control vs. FMT-Healthy	-0,3333	-1,045 to 0,3786	No	ns	0,5114
FMT-CCR vs. FMT-Healthy	-0,3333	-1,045 to 0,3786	No	ns	0,5114
Glandular hyperplasia					
Positive Control vs. FMT-CCR	0	-0,7119 to 0,7119	No	ns	>0,9999
Positive Control vs. FMT-Healthy	0	-0,7119 to 0,7119	No	ns	>0,9999
FMT-CCR vs. FMT-Healthy	0	-0,7119 to 0,7119	No	ns	>0,9999
Mitosis					
Positive Control vs. FMT-CCR	-1,667	-2,379 to -0,9547	Yes	****	<0,0001
Positive Control vs. FMT-Healthy	-1,667	-2,379 to -0,9547	Yes	****	<0,0001
FMT-CCR vs. FMT-Healthy	0	-0,7119 to 0,7119	No	ns	>0,9999
Apoptosis					
Positive Control vs. FMT-CCR	0,6667	-0,04527 to 1,379	No	ns	0,0717
Positive Control vs. FMT-Healthy	0	-0,7119 to 0,7119	No	ns	>0,9999
FMT-CCR vs. FMT-Healthy	-0,6667	-1,379 to 0,04527	No	ns	0,0717
Aberrant crypts					
Positive Control vs. FMT-CCR	-0,3333	-1,045 to 0,3786	No	ns	0,5114
Positive Control vs. FMT-Healthy	-0,6667	-1,379 to 0,04527	No	ns	0,0717
FMT-CCR vs. FMT-Healthy	-0,3333	-1,045 to 0,3786	No	ns	0,5114
Epithelial dysplasia					
Positive Control vs. FMT-CCR	0	-0,7119 to 0,7119	No	ns	>0,9999
Positive Control vs. FMT-Healthy	0	-0,7119 to 0,7119	No	ns	>0,9999
FMT-CCR vs. FMT-Healthy	0	-0,7119 to 0,7119	No	ns	>0,9999
Glandular dysplasia					
Positive Control vs. FMT-CCR	0	-0,7119 to 0,7119	No	ns	>0,9999
Positive Control vs. FMT-Healthy	0	-0,7119 to 0,7119	No	ns	>0,9999
FMT-CCR vs. FMT-Healthy	0	-0,7119 to 0,7119	No	ns	>0,9999
Neoplastic proliferation					
Positive Control vs. FMT-CCR	-1,333	-2,045 to -0,6214	Yes	****	<0,0001
Positive Control vs. FMT-Healthy	-1,667	-2,379 to -0,9547	Yes	****	<0,0001
FMT-CCR vs. FMT-Healthy	-0,3333	-1,045 to 0,3786	No	ns	0,5114
Neoplastic infiltration					
Positive Control vs. FMT-CCR	0	-0,7119 to 0,7119	No	ns	>0,9999
Positive Control vs. FMT-Healthy	0	-0,7119 to 0,7119	No	ns	>0,9999
FMT-CCR vs. FMT-Healthy	0	-0,7119 to 0,7119	No	ns	>0,9999
GALT hyperplasia					

Positive Control vs. FMT-CCR	-0,6667	-1,379 to 0,04527	No	ns	0,0717
Positive Control vs. FMT-Healthy	0,3333	-0,3786 to 1,045	No	ns	0,5114
FMT-CCR vs. FMT-Healthy	1	0,2881 to 1,712	Yes	**	0,0031
Erosion					
Positive Control vs. FMT-CCR	0	-0,7119 to 0,7119	No	ns	>0,9999
Positive Control vs. FMT-Healthy	0	-0,7119 to 0,7119	No	ns	>0,9999
FMT-CCR vs. FMT-Healthy	0	-0,7119 to 0,7119	No	ns	>0,9999
Ulceration					
Positive Control vs. FMT-CCR	0	-0,7119 to 0,7119	No	ns	>0,9999
Positive Control vs. FMT-Healthy	0	-0,7119 to 0,7119	No	ns	>0,9999
FMT-CCR vs. FMT-Healthy	0	-0,7119 to 0,7119	No	ns	>0,9999
Pigment					
Positive Control vs. FMT-CCR	0	-0,7119 to 0,7119	No	ns	>0,9999
Positive Control vs. FMT-Healthy	0	-0,7119 to 0,7119	No	ns	>0,9999
FMT-CCR vs. FMT-Healthy	0	-0,7119 to 0,7119	No	ns	>0,9999
Minerals					
Positive Control vs. FMT-CCR	0	-0,7119 to 0,7119	No	ns	>0,9999
Positive Control vs. FMT-Healthy	0	-0,7119 to 0,7119	No	ns	>0,9999
FMT-CCR vs. FMT-Healthy	0	-0,7119 to 0,7119	No	ns	>0,9999
Necrosis					
Positive Control vs. FMT-CCR	-0,6667	-1,379 to 0,04527	No	ns	0,0717
Positive Control vs. FMT-Healthy	-0,6667	-1,379 to 0,04527	No	ns	0,0717
FMT-CCR vs. FMT-Healthy	0	-0,7119 to 0,7119	No	ns	>0,9999
Congestion					
Positive Control vs. FMT-CCR	-0,3333	-1,045 to 0,3786	No	ns	0,5114
Positive Control vs. FMT-Healthy	-0,6667	-1,379 to 0,04527	No	ns	0,0717
FMT-CCR vs. FMT-Healthy	-0,3333	-1,045 to 0,3786	No	ns	0,5114
Edema					
Positive Control vs. FMT-CCR	0	-0,7119 to 0,7119	No	ns	>0,9999
Positive Control vs. FMT-Healthy	0	-0,7119 to 0,7119	No	ns	>0,9999
FMT-CCR vs. FMT-Healthy	0	-0,7119 to 0,7119	No	ns	>0,9999
Hemorrhage					
Positive Control vs. FMT-CCR	0	-0,7119 to 0,7119	No	ns	>0,9999
Positive Control vs. FMT-Healthy	0	-0,7119 to 0,7119	No	ns	>0,9999
FMT-CCR vs. FMT-Healthy	0	-0,7119 to 0,7119	No	ns	>0,9999

Thrombosis					
Positive Control vs. FMT-CCR	0	-0,7119 to 0,7119	No	ns	>0,9999
Positive Control vs. FMT-Healthy	0	-0,7119 to 0,7119	No	ns	>0,9999
FMT-CCR vs. FMT-Healthy	0	-0,7119 to 0,7119	No	ns	>0,9999
Fibrin					
Positive Control vs. FMT-CCR	0	-0,7119 to 0,7119	No	ns	>0,9999
Positive Control vs. FMT-Healthy	0	-0,7119 to 0,7119	No	ns	>0,9999
FMT-CCR vs. FMT-Healthy	0	-0,7119 to 0,7119	No	ns	>0,9999
Exocytosis					
Positive Control vs. FMT-CCR	0	-0,7119 to 0,7119	No	ns	>0,9999
Positive Control vs. FMT-Healthy	0	-0,7119 to 0,7119	No	ns	>0,9999
FMT-CCR vs. FMT-Healthy	0	-0,7119 to 0,7119	No	ns	>0,9999
Neutrophils					
Positive Control vs. FMT-CCR	0	-0,7119 to 0,7119	No	ns	>0,9999
Positive Control vs. FMT-Healthy	0	-0,7119 to 0,7119	No	ns	>0,9999
FMT-CCR vs. FMT-Healthy	0	-0,7119 to 0,7119	No	ns	>0,9999
Eosinophils					
Positive Control vs. FMT-CCR	0,3333	-0,3786 to 1,045	No	ns	0,5114
Positive Control vs. FMT-Healthy	0,3333	-0,3786 to 1,045	No	ns	0,5114
FMT-CCR vs. FMT-Healthy	-1,11E-16	-0,7119 to 0,7119	No	ns	>0,9999
Macrophages					
Positive Control vs. FMT-CCR	0	-0,7119 to 0,7119	No	ns	>0,9999
Positive Control vs. FMT-Healthy	0	-0,7119 to 0,7119	No	ns	>0,9999
FMT-CCR vs. FMT-Healthy	0	-0,7119 to 0,7119	No	ns	>0,9999
Lymphocytes					
Positive Control vs. FMT-CCR	-0,3333	-1,045 to 0,3786	No	ns	0,5114
Positive Control vs. FMT-Healthy	0	-0,7119 to 0,7119	No	ns	>0,9999
FMT-CCR vs. FMT-Healthy	0,3333	-0,3786 to 1,045	No	ns	0,5114
Plasma cells					
Positive Control vs. FMT-CCR	-0,3333	-1,045 to 0,3786	No	ns	0,5114
Positive Control vs. FMT-Healthy	0	-0,7119 to 0,7119	No	ns	>0,9999
FMT-CCR vs. FMT-Healthy	0,3333	-0,3786 to 1,045	No	ns	0,5114
Fibrosis					
Positive Control vs. FMT-CCR	0	-0,7119 to 0,7119	No	ns	>0,9999
Positive Control vs. FMT-Healthy	0	-0,7119 to 0,7119	No	ns	>0,9999
FMT-CCR vs. FMT-Healthy	0	-0,7119 to 0,7119	No	ns	>0,9999

Biological agents								
Positive Control vs. FMT-CCR	0	-0,7119 to 0,7119	No	ns	>0,9999			
Positive Control vs. FMT-Healthy	0	-0,7119 to 0,7119	No	ns	>0,9999			
FMT-CCR vs. FMT-Healthy	0	-0,7119 to 0,7119	No	ns	>0,9999			

Test details	Mean 1	Mean 2	Mean Diff,	SE of diff,	N1	N2	q	DF
Epithelial atrophy								
Positive Control vs. FMT-CCR	-2,22E-16	-2,22E-16	0	0,3013	3	3	0	186
Positive Control vs. FMT-Healthy	-2,22E-16	0	-2,22E-16	0,3013	3	3	1,042e-015	186
FMT-CCR vs. FMT-Healthy	-2,22E-16	0	-2,22E-16	0,3013	3	3	1,042e-015	186
Glandular atrophy								
Positive Control vs. FMT-CCR	0	0	0	0,3013	3	3	0	186
Positive Control vs. FMT-Healthy	0	0	0	0,3013	3	3	0	186
FMT-CCR vs. FMT-Healthy	0	0	0	0,3013	3	3	0	186
Goblet cell								
Positive Control vs. FMT-CCR	0,6667	0	0,6667	0,3013	3	3	3,129	186
Positive Control vs. FMT-Healthy	0,6667	0,3333	0,3333	0,3013	3	3	1,564	186
FMT-CCR vs. FMT-Healthy	0	0,3333	-0,3333	0,3013	3	3	1,564	186
Epithelial hyperplasia								
Positive Control vs. FMT-CCR	0,3333	0,3333	0	0,3013	3	3	0	186
Positive Control vs. FMT-Healthy	0,3333	0,6667	-0,3333	0,3013	3	3	1,564	186
FMT-CCR vs. FMT-Healthy	0,3333	0,6667	-0,3333	0,3013	3	3	1,564	186
Glandular hyperplasia								
Positive Control vs. FMT-CCR	0,6667	0,6667	0	0,3013	3	3	0	186
Positive Control vs. FMT-Healthy	0,6667	0,6667	0	0,3013	3	3	0	186
FMT-CCR vs. FMT-Healthy	0,6667	0,6667	0	0,3013	3	3	0	186
Mitosis								
Positive Control vs. FMT-CCR	1	2,667	-1,667	0,3013	3	3	7,822	186
Positive Control vs. FMT-Healthy	1	2,667	-1,667	0,3013	3	3	7,822	186
FMT-CCR vs. FMT-Healthy	2,667	2,667	0	0,3013	3	3	0	186
Apoptosis								
Positive Control vs. FMT-CCR	0,6667	0	0,6667	0,3013	3	3	3,129	186
Positive Control vs. FMT-Healthy	0,6667	0,6667	0	0,3013	3	3	0	186
FMT-CCR vs. FMT-Healthy	0	0,6667	-0,6667	0,3013	3	3	3,129	186

Aberrant crypts								
Positive Control vs. FMT-CCR	0,3333	0,6667	-0,3333	0,3013	3	3	1,564	186
Positive Control vs. FMT-Healthy	0,3333	1	-0,6667	0,3013	3	3	3,129	186
FMT-CCR vs. FMT-Healthy	0,6667	1	-0,3333	0,3013	3	3	1,564	186
Epithelial dysplasia								
Positive Control vs. FMT-CCR	0	0	0	0,3013	3	3	0	186
Positive Control vs. FMT-Healthy	0	0	0	0,3013	3	3	0	186
FMT-CCR vs. FMT-Healthy	0	0	0	0,3013	3	3	0	186
Glandular dysplasia								
Positive Control vs. FMT-CCR	0,3333	0,3333	0	0,3013	3	3	0	186
Positive Control vs. FMT-Healthy	0,3333	0,3333	0	0,3013	3	3	0	186
FMT-CCR vs. FMT-Healthy	0,3333	0,3333	0	0,3013	3	3	0	186
Neoplastic proliferation								
Positive Control vs. FMT-CCR	1,333	2,667	-1,333	0,3013	3	3	6,258	186
Positive Control vs. FMT-Healthy	1,333	3	-1,667	0,3013	3	3	7,822	186
FMT-CCR vs. FMT-Healthy	2,667	3	-0,3333	0,3013	3	3	1,564	186
Neoplastic infiltration								
Positive Control vs. FMT-CCR	1	1	0	0,3013	3	3	0	186
Positive Control vs. FMT-Healthy	1	1	0	0,3013	3	3	0	186
FMT-CCR vs. FMT-Healthy	1	1	0	0,3013	3	3	0	186
GALT hyperplasia								
Positive Control vs. FMT-CCR	1,333	2	-0,6667	0,3013	3	3	3,129	186
Positive Control vs. FMT-Healthy	1,333	1	0,3333	0,3013	3	3	1,564	186
FMT-CCR vs. FMT-Healthy	2	1	1	0,3013	3	3	4,693	186
Erosion								
Positive Control vs. FMT-CCR	0	0	0	0,3013	3	3	0	186
Positive Control vs. FMT-Healthy	0	0	0	0,3013	3	3	0	186
FMT-CCR vs. FMT-Healthy	0	0	0	0,3013	3	3	0	186
Ulceration								
Positive Control vs. FMT-CCR	0	0	0	0,3013	3	3	0	186
Positive Control vs. FMT-Healthy	0	0	0	0,3013	3	3	0	186
FMT-CCR vs. FMT-Healthy	0	0	0	0,3013	3	3	0	186
Pigment								
Positive Control vs. FMT-CCR	0	0	0	0,3013	3	3	0	186
Positive Control vs. FMT-Healthy	0	0	0	0,3013	3	3	0	186
FMT-CCR vs. FMT-Healthy	0	0	0	0,3013	3	3	0	186

Minerals								
Positive Control vs. FMT-CCR	0	0	0	0,3013	3	3	0	186
Positive Control vs. FMT-Healthy	0	0	0	0,3013	3	3	0	186
FMT-CCR vs. FMT-Healthy	0	0	0	0,3013	3	3	0	186
Necrosis								
Positive Control vs. FMT-CCR	0,3333	1	-0,6667	0,3013	3	3	3,129	186
Positive Control vs. FMT-Healthy	0,3333	1	-0,6667	0,3013	3	3	3,129	186
FMT-CCR vs. FMT-Healthy	1	1	0	0,3013	3	3	0	186
Congestion								
Positive Control vs. FMT-CCR	0,3333	0,6667	-0,3333	0,3013	3	3	1,564	186
Positive Control vs. FMT-Healthy	0,3333	1	-0,6667	0,3013	3	3	3,129	186
FMT-CCR vs. FMT-Healthy	0,6667	1	-0,3333	0,3013	3	3	1,564	186
Edema								
Positive Control vs. FMT-CCR	0	0	0	0,3013	3	3	0	186
Positive Control vs. FMT-Healthy	0	0	0	0,3013	3	3	0	186
FMT-CCR vs. FMT-Healthy	0	0	0	0,3013	3	3	0	186
Hemorrhage								
Positive Control vs. FMT-CCR	0	0	0	0,3013	3	3	0	186
Positive Control vs. FMT-Healthy	0	0	0	0,3013	3	3	0	186
FMT-CCR vs. FMT-Healthy	0	0	0	0,3013	3	3	0	186
Thrombosis								
Positive Control vs. FMT-CCR	0	0	0	0,3013	3	3	0	186
Positive Control vs. FMT-Healthy	0	0	0	0,3013	3	3	0	186
FMT-CCR vs. FMT-Healthy	0	0	0	0,3013	3	3	0	186
Fibrin								
Positive Control vs. FMT-CCR	0	0	0	0,3013	3	3	0	186
Positive Control vs. FMT-Healthy	0	0	0	0,3013	3	3	0	186
FMT-CCR vs. FMT-Healthy	0	0	0	0,3013	3	3	0	186
Exocytosis								
Positive Control vs. FMT-CCR	0	0	0	0,3013	3	3	0	186
Positive Control vs. FMT-Healthy	0	0	0	0,3013	3	3	0	186
FMT-CCR vs. FMT-Healthy	0	0	0	0,3013	3	3	0	186
Neutrophils								
Positive Control vs. FMT-CCR	1	1	0	0,3013	3	3	0	186
Positive Control vs. FMT-Healthy	1	1	0	0,3013	3	3	0	186

FMT-CCR vs. FMT-Healthy	1	1	0	0,3013	3	3	0	186
Eosinophils								
Positive Control vs. FMT-CCR	0,3333	-3,33E-16	0,3333	0,3013	3	3	1,564	186
Positive Control vs. FMT-Healthy	0,3333	-2,22E-16	0,3333	0,3013	3	3	1,564	186
FMT-CCR vs. FMT-Healthy	-3,33E-16	-2,22E-16	-1,11E-16	0,3013	3	3	5,211e-016	186
Macrophages								
Positive Control vs. FMT-CCR	1	1	0	0,3013	3	3	0	186
Positive Control vs. FMT-Healthy	1	1	0	0,3013	3	3	0	186
FMT-CCR vs. FMT-Healthy	1	1	0	0,3013	3	3	0	186
Lymphocytes								
Positive Control vs. FMT-CCR	1	1,333	-0,3333	0,3013	3	3	1,564	186
Positive Control vs. FMT-Healthy	1	1	0	0,3013	3	3	0	186
FMT-CCR vs. FMT-Healthy	1,333	1	0,3333	0,3013	3	3	1,564	186
Plasma cells								
Positive Control vs. FMT-CCR	1	1,333	-0,3333	0,3013	3	3	1,564	186
Positive Control vs. FMT-Healthy	1	1	0	0,3013	3	3	0	186
FMT-CCR vs. FMT-Healthy	1,333	1	0,3333	0,3013	3	3	1,564	186
Fibrosis								
Positive Control vs. FMT-CCR	1	1	0	0,3013	3	3	0	186
Positive Control vs. FMT-Healthy	1	1	0	0,3013	3	3	0	186
FMT-CCR vs. FMT-Healthy	1	1	0	0,3013	3	3	0	186
Biological agents								
Positive Control vs. FMT-CCR	0	0	0	0,3013	3	3	0	186
Positive Control vs. FMT-Healthy	0	0	0	0,3013	3	3	0	186
FMT-CCR vs. FMT-Healthy	0	0	0	0,3013	3	3	0	186

Supplementary material

S7:

Table Analyzed	IL-1β				
Two-way ANOVA	Ordinary				
Alpha	0,05				
Source of Variation	% of total variation	P value	P value summ	Significant?	
Interaction	15,59	0,327	ns	No	
Row Factor	3,313	0,3859	ns	No	
Column Factor	22,04	0,1977	ns	No	
ANOVA table	SS (Type III)	DF	MS	F (DFn, DFd)	P value
Interaction	4349	3	1450	F (3, 14) = 1,2	P=0,3270
Row Factor	923,8	1	923,8	F (1, 14) = 0,8	P=0,3859
Column Factor	6148	3	2049	F (3, 14) = 1,7	P=0,1977
Residual	16147	14	1153		
Difference between row means					
Predicted (LS) mean of Week 5	33,49				
Predicted (LS) mean of Week 12	46,65				
Difference between predicted means	-13,16				
SE of difference	14,71				
95% CI of difference	-44,70 to 18,38				
Within each row, compare columns (simple effects within rows)					
Number of families	2				
Number of comparisons per family	6				
Alpha	0,05				
Tukey's multiple comparisons test	Mean Diff,	95,00% CI of diff,	Significant?	Summary	Adjusted P Value
Week 5					
Negative Control vs. Positive Control	-11,22	-101,3 to 78,89	No	ns	0,9831
Negative Control vs. FMT-CCR	-36,12	-126,2 to 54,00	No	ns	0,6574
Negative Control vs. FMT-Healthy	-41,01	-131,1 to 49,10	No	ns	0,5643
Positive Control vs. FMT-CCR	-24,9	-105,5 to 55,70	No	ns	0,8062
Positive Control vs. FMT-Healthy	-29,79	-110,4 to 50,80	No	ns	0,71
FMT-CCR vs. FMT-Healthy	-4,898	-85,50 to 75,70	No	ns	0,9979
Week 12					
Negative Control vs. Positive Control	-79,07	-169,2 to 11,04	No	ns	0,0947

Negative Control vs. FMT-CCR	-48,46	-138,6 to 41,65	No	ns	0,4292
Negative Control vs. FMT-Healthy	-43,47	-133,6 to 46,64	No	ns	0,5184
Positive Control vs. FMT-CCR	30,61	-49,98 to 111,2	No	ns	0,693
Positive Control vs. FMT-Healthy	35,6	-44,99 to 116,2	No	ns	0,5872
FMT-CCR vs. FMT-Healthy	4,99	-75,61 to 85,59	No	ns	0,9978

Test details	Mean 1	Mean 2	Mean Diff,	SE of diff,	N1	N2	q	DF
Week 5								
Negative Control vs. Positive Control	11,4	22,62	-11,22	31	1	1	0,5118	14
Negative Control vs. FMT-CCR	11,4	47,52	-36,12	31	1	1	1,647	14
Negative Control vs. FMT-Healthy	11,4	52,41	-41,01	31	1	1	1,871	14
Positive Control vs. FMT-CCR	22,62	47,52	-24,9	27,73	1	1	1,27	14
Positive Control vs. FMT-Healthy	22,62	52,41	-29,79	27,73	1	1	1,519	14
FMT-CCR vs. FMT-Healthy	47,52	52,41	-4,898	27,73	1	1	0,2498	14
Week 12								
Negative Control vs. Positive Control	3,9	82,97	-79,07	31	1	1	3,607	14
Negative Control vs. FMT-CCR	3,9	52,36	-48,46	31	1	1	2,21	14
Negative Control vs. FMT-Healthy	3,9	47,37	-43,47	31	1	1	1,983	14
Positive Control vs. FMT-CCR	82,97	52,36	30,61	27,73	1	1	1,561	14
Positive Control vs. FMT-Healthy	82,97	47,37	35,6	27,73	1	1	1,816	14
FMT-CCR vs. FMT-Healthy	52,36	47,37	4,99	27,73	1	1	0,2545	14

Supplementary material

S8:

Table Analyzed	IL-2				
Two-way ANOVA	Ordinary				
Alpha	0,05				
Source of Variation	% of total variation	P value	P value summary	Significant?	
Interaction	18,24	0,0011	**	Yes	
Row Factor	0,7944	0,2825	ns	No	
Column Factor	72,83	<0,0001	****	Yes	
ANOVA table	SS (Type III)	DF	MS	F (DFn, DFd)	P value
Interaction	184,9	3	61,63	F (3, 14) = 9,559	P=0,0011
Row Factor	8,054	1	8,054	F (1, 14) = 1,249	P=0,2825
Column Factor	738,4	3	246,1	F (3, 14) = 38,17	P<0,0001
Residual	90,26	14	6,447		
Difference between row means					
Predicted (LS) mean of Week 5	6,414				
Predicted (LS) mean of Week 12	7,643				
Difference between predicted means	-1,229				
SE of difference	1,099				
95% CI of difference	-3,587 to 1,129				
Within each row, compare columns (simple effects within rows)					
Number of families	2				
Number of comparisons per family	6				
Alpha	0,05				

Tukey's multiple comparisons test	Mean Diff,	95,00% CI of diff,	Significant?	Summary	Adjusted P Value
Week 5					
Negative Control vs. Positive Control	7,64	0,9028 to 14,38	Yes	*	0,0242
Negative Control vs. FMT-CCR	8,387	1,650 to 15,12	Yes	*	0,0132
Negative Control vs. FMT-Healthy	6,437	-0,3005 to 13,17	No	ns	0,0633
Positive Control vs. FMT-CCR	0,7469	-5,279 to 6,773	No	ns	0,9833
Positive Control vs. FMT-Healthy	-1,203	-7,229 to 4,823	No	ns	0,9363
FMT-CCR vs. FMT-Healthy	-1,95	-7,976 to 4,076	No	ns	0,7839
Week 12					
Negative Control vs. Positive Control	23,04	16,30 to 29,78	Yes	****	<0,0001

Negative Control vs. FMT-CCR	22,38	15,64 to 29,11	Yes	****	<0,0001
Negative Control vs. FMT-Healthy	21,89	15,16 to 28,63	Yes	****	<0,0001
Positive Control vs. FMT-CCR	-0,6641	-6,690 to 5,362	No	ns	0,9881
Positive Control vs. FMT-Healthy	-1,148	-7,174 to 4,878	No	ns	0,944
FMT-CCR vs. FMT-Healthy	-0,4836	-6,509 to 5,542	No	ns	0,9953

Test details	Mean 1	Mean 2	Mean Diff,	SE of diff,	N1	N2	q	DF
Week 5								
Negative Control vs. Positive Control	12,03	4,39	7,64	2,318	1	1	4,661	14
Negative Control vs. FMT-CCR	12,03	3,643	8,387	2,318	1	1	5,117	14
Negative Control vs. FMT-Healthy	12,03	5,593	6,437	2,318	1	1	3,927	14
Positive Control vs. FMT-CCR	4,39	3,643	0,7469	2,073	1	1	0,5095	14
Positive Control vs. FMT-Healthy	4,39	5,593	-1,203	2,073	1	1	0,8208	14
FMT-CCR vs. FMT-Healthy	3,643	5,593	-1,95	2,073	1	1	1,33	14
Week 12								
Negative Control vs. Positive Control	24,47	1,43	23,04	2,318	1	1	14,06	14
Negative Control vs. FMT-CCR	24,47	2,094	22,38	2,318	1	1	13,65	14
Negative Control vs. FMT-Healthy	24,47	2,578	21,89	2,318	1	1	13,36	14
Positive Control vs. FMT-CCR	1,43	2,094	-0,6641	2,073	1	1	0,453	14
Positive Control vs. FMT-Healthy	1,43	2,578	-1,148	2,073	1	1	0,7829	14
FMT-CCR vs. FMT-Healthy	2,094	2,578	-0,4836	2,073	1	1	0,3299	14

Supplementary material

S9:

Table Analyzed	IL-4				
Two-way ANOVA	Ordinary				
Alpha	0,05				

Source of Variation	% of total varia	P value	P value summary	Significant?
Interaction	5,474	0,7781	ns	No
Row Factor	8,059	0,2238	ns	No
Column Factor	15,63	0,4024	ns	No

ANOVA table	SS (Type III)	DF	MS	F (DFn, DFd)	P value
Interaction	24,58	3	8,193	F (3, 14) = 0,3668	P=0,7781
Row Factor	36,19	1	36,19	F (1, 14) = 1,620	P=0,2238
Column Factor	70,17	3	23,39	F (3, 14) = 1,047	P=0,4024
Residual	312,7	14	22,34		

Difference between row means	
Predicted (LS) mean of Week 5	6,003
Predicted (LS) mean of Week 12	3,399
Difference between predicted means	2,605
SE of difference	2,046
95% CI of difference	-1,784 to 6,994

Within each row, compare columns (simple effects within rows)

Number of families	2
Number of comparisons per family	6
Alpha	0,05

Tukey's multiple comparisons test	Mean Diff,	95,00% CI of diff,	Significant?	Summary	Adjusted P Value
Week 5					
Negative Control vs. Positive Control	-4,04	-16,58 to 8,500	No	ns	0,7862
Negative Control vs. FMT-CCR	-4,681	-17,22 to 7,858	No	ns	0,7039
Negative Control vs. FMT-Healthy	-6,612	-19,15 to 5,928	No	ns	0,4455
Positive Control vs. FMT-CCR	-0,6415	-11,86 to 10,57	No	ns	0,9983
Positive Control vs. FMT-Healthy	-2,572	-13,79 to 8,644	No	ns	0,9079
FMT-CCR vs. FMT-Healthy	-1,931	-13,15 to 9,285	No	ns	0,9577
Week 12					
Negative Control vs. Positive Control	-1,49	-14,03 to 11,05	No	ns	0,9852

Negative Control vs. FMT-CCR	-5,401	-17,94 to 7,139	No	ns	0,6062
Negative Control vs. FMT-Healthy	-2,104	-14,64 to 10,44	No	ns	0,9606
Positive Control vs. FMT-CCR	-3,911	-15,13 to 7,305	No	ns	0,7444
Positive Control vs. FMT-Healthy	-0,6137	-11,83 to 10,60	No	ns	0,9985
FMT-CCR vs. FMT-Healthy	3,297	-7,919 to 14,51	No	ns	0,8277

Test details	Mean 1	Mean 2	Mean Diff,	SE of diff,	N1	N2	q	DF
Week 5								
Negative Control vs. Positive Control	2,17	6,21	-4,04	4,314	1	1	1,324	14
Negative Control vs. FMT-CCR	2,17	6,851	-4,681	4,314	1	1	1,535	14
Negative Control vs. FMT-Healthy	2,17	8,782	-6,612	4,314	1	1	2,167	14
Positive Control vs. FMT-CCR	6,21	6,851	-0,6415	3,859	1	1	0,2351	14
Positive Control vs. FMT-Healthy	6,21	8,782	-2,572	3,859	1	1	0,9426	14
FMT-CCR vs. FMT-Healthy	6,851	8,782	-1,931	3,859	1	1	0,7075	14
Week 12								
Negative Control vs. Positive Control	1,15	2,64	-1,49	4,314	1	1	0,4884	14
Negative Control vs. FMT-CCR	1,15	6,551	-5,401	4,314	1	1	1,77	14
Negative Control vs. FMT-Healthy	1,15	3,254	-2,104	4,314	1	1	0,6896	14
Positive Control vs. FMT-CCR	2,64	6,551	-3,911	3,859	1	1	1,433	14
Positive Control vs. FMT-Healthy	2,64	3,254	-0,6137	3,859	1	1	0,2249	14
FMT-CCR vs. FMT-Healthy	6,551	3,254	3,297	3,859	1	1	1,208	14

Supplementary material

S10:

Table Analyzed	IL-6				
Two-way ANOVA	Ordinary				
Alpha	0,05				
Source of Variation	% of total variation	P value	P value summary	Significant?	
Interaction	17	0,2627	ns	No	
Row Factor	4,218	0,3117	ns	No	
Column Factor	24,22	0,145	ns	No	
ANOVA table	SS (Type III)	DF	MS	F (DFn, DFd)	P value
Interaction	445430189	3	148476730	F (3, 14) = 1,480	P=0,2627
Row Factor	110496678	1	110496678	F (1, 14) = 1,102	P=0,3117
Column Factor	634502117	3	211500706	F (3, 14) = 2,109	P=0,1450
Residual	1404106109	14	100293293		
Difference between row means					
Predicted (LS) mean of Week 5	1092				
Predicted (LS) mean of Week 12	5644				
Difference between predicted means	-4552				
SE of difference	4336				
95% CI of difference	-13853 to 4749				
Within each row, compare columns (simple effects within rows)					
Number of families	2				
Number of comparisons per family	6				
Alpha	0,05				
Tukey's multiple comparisons test	Mean Diff,	95,00% CI of diff,	Significant?	Summary	Adjusted P Value
Week 5					
Negative Control vs. Positive Control	-9,78	-26582 to 26562	No	ns	>0,9999
Negative Control vs. FMT-CCR	-1696	-28268 to 24877	No	ns	0,9976
Negative Control vs. FMT-Healthy	-2591	-29164 to 23981	No	ns	0,9917
Positive Control vs. FMT-CCR	-1686	-25453 to 22081	No	ns	0,9968
Positive Control vs. FMT-Healthy	-2582	-26348 to 21185	No	ns	0,9886
FMT-CCR vs. FMT-Healthy	-895,9	-24663 to 22871	No	ns	0,9995
Week 12					
Negative Control vs. Positive Control	-72,72	-26645 to 26499	No	ns	>0,9999

Negative Control vs. FMT-CCR	-166,6	-26739 to 26405	No	ns	>0,9999
Negative Control vs. FMT-Healthy	-22196	-48768 to 4376	No	ns	0,117
Positive Control vs. FMT-CCR	-93,89	-23861 to 23673	No	ns	>0,9999
Positive Control vs. FMT-Healthy	-22123	-45890 to 1643	No	ns	0,072
FMT-CCR vs. FMT-Healthy	-22029	-45796 to 1737	No	ns	0,0735

Test details	Mean 1	Mean 2	Mean Diff,	SE of diff,	N1	N2	q	DF
Week 5								
Negative Control vs. Positive Control	17,71	27,49	-9,78	9142	1	1	0,001513	14
Negative Control vs. FMT-CCR	17,71	1713	-1696	9142	1	1	0,2623	14
Negative Control vs. FMT-Healthy	17,71	2609	-2591	9142	1	1	0,4009	14
Positive Control vs. FMT-CCR	27,49	1713	-1686	8177	1	1	0,2916	14
Positive Control vs. FMT-Healthy	27,49	2609	-2582	8177	1	1	0,4465	14
FMT-CCR vs. FMT-Healthy	1713	2609	-895,9	8177	1	1	0,1549	14
Week 12								
Negative Control vs. Positive Control	34,78	107,5	-72,72	9142	1	1	0,01125	14
Negative Control vs. FMT-CCR	34,78	201,4	-166,6	9142	1	1	0,02577	14
Negative Control vs. FMT-Healthy	34,78	22231	-22196	9142	1	1	3,434	14
Positive Control vs. FMT-CCR	107,5	201,4	-93,89	8177	1	1	0,01624	14
Positive Control vs. FMT-Healthy	107,5	22231	-22123	8177	1	1	3,826	14
FMT-CCR vs. FMT-Healthy	201,4	22231	-22029	8177	1	1	3,81	14

Supplementary material

S11:

Table Analyzed	IL-10				
Two-way ANOVA	Ordinary				
Alpha	0,05				
Source of Variation	% of total variation	P value	P value summary	Significant?	
Interaction	22,66	0,1378	ns	No	
Row Factor	5,204	0,2422	ns	No	
Column Factor	22,31	0,1421	ns	No	
ANOVA table	SS (Type III)	DF	MS	F (DFn, DFd)	P value
Interaction	47092	3	15697	F (3, 14) = 2,164	P=0,1378
Row Factor	10816	1	10816	F (1, 14) = 1,491	P=0,2422
Column Factor	46375	3	15458	F (3, 14) = 2,131	P=0,1421
Residual	101542	14	7253		
Difference between row means					
Predicted (LS) mean of Week 5	68,44				
Predicted (LS) mean of Week 12	23,4				
Difference between predicted means	45,03				
SE of difference	36,88				
95% CI of difference	-34,06 to 124,1				
Within each row, compare columns (simple effects within rows)					
Number of families	2				
Number of comparisons per family	6				
Alpha	0,05				
Tukey's multiple comparisons test	Mean Diff,	95,00% CI of d	Significant?	Summary	Adjusted P Value
Week 5					
Negative Control vs. Positive Control	11,56	214,4 to 237,5	No	ns	0,9988
Negative Control vs. FMT-CCR	1,019	224,9 to 227,0	No	ns	>0,9999
Negative Control vs. FMT-Healthy	-198,7	424,7 to 27,25	No	ns	0,0938
Positive Control vs. FMT-CCR	-10,54	212,7 to 191,6	No	ns	0,9987
Positive Control vs. FMT-Healthy	-210,3	[illegible]12,4 to -8,167	Yes	*	0,0403
FMT-CCR vs. FMT-Healthy	-199,7	401,9 to 2,374	No	ns	0,0532
Week 12					
Negative Control vs. Positive Control	5,02	220,9 to 231,0	No	ns	0,9999

Negative Control vs. FMT-CCR	-36,35	262,3 to 189,6	No	ns	0,965
Negative Control vs. FMT-Healthy	-9,799	235,8 to 216,2	No	ns	0,9992
Positive Control vs. FMT-CCR	-41,37	243,5 to 160,7	No	ns	0,9319
Positive Control vs. FMT-Healthy	-14,82	216,9 to 187,3	No	ns	0,9964
FMT-CCR vs. FMT-Healthy	26,55	175,6 to 228,7	No	ns	0,9803

Test details	Mean 1	Mean 2	Mean Diff,	SE of diff,	N1	N2	q	DF
Week 5								
Negative Control vs. Positive Control	21,9	10,34	11,56	77,74	1	1	0,2103	14
Negative Control vs. FMT-CCR	21,9	20,88	1,019	77,74	1	1	0,01854	14
Negative Control vs. FMT-Healthy	21,9	220,6	-198,7	77,74	1	1	3,615	14
Positive Control vs. FMT-CCR	10,34	20,88	-10,54	69,54	1	1	0,2144	14
Positive Control vs. FMT-Healthy	10,34	220,6	-210,3	69,54	1	1	4,277	14
FMT-CCR vs. FMT-Healthy	20,88	220,6	-199,7	69,54	1	1	4,062	14
Week 12								
Negative Control vs. Positive Control	13,12	8,1	5,02	77,74	1	1	0,09132	14
Negative Control vs. FMT-CCR	13,12	49,47	-36,35	77,74	1	1	0,6612	14
Negative Control vs. FMT-Healthy	13,12	22,92	-9,799	77,74	1	1	0,1783	14
Positive Control vs. FMT-CCR	8,1	49,47	-41,37	69,54	1	1	0,8413	14
Positive Control vs. FMT-Healthy	8,1	22,92	-14,82	69,54	1	1	0,3014	14
FMT-CCR vs. FMT-Healthy	49,47	22,92	26,55	69,54	1	1	0,5399	14

Supplementary material

S12:

Table Analyzed	IL-17A				
Two-way ANOVA	Ordinary				
Alpha	0,05				
Source of Variation	% of total variation	P value	P value summary	Significant?	
Interaction	7,677	0,4401	ns	No	
Row Factor	3,797	0,2532	ns	No	
Column Factor	49,75	0,0067	**	Yes	
ANOVA table	SS (Type III)	DF	MS	F (DFn, DFd)	P value
Interaction	7623	3	2541	F (3, 14) = 0,9570	P=0,4401
Row Factor	3770	1	3770	F (1, 14) = 1,420	P=0,2532
Column Factor	49401	3	16467	F (3, 14) = 6,202	P=0,0067
Residual	37175	14	2655		
Difference between row means					
Predicted (LS) mean of Week 5	67,04				
Predicted (LS) mean of Week 12	93,63				
Difference between predicted means	-26,59				
SE of difference	22,31				
95% CI of difference	-74,45 to 21,27				

Supplementary material

S13:

Table Analyzed	TNFα				
Two-way ANOVA	Ordinary				
Alpha	0,05				
Source of Variation	% of total variation	P value	P value summ	Significant?	
Interaction	17,92	0,2605	ns	No	
Row Factor	0,5042	0,7282	ns	No	
Column Factor	25,33	0,1454	ns	No	
ANOVA table	SS (Type III)	DF	MS	F (DFn, DFd)	P value
Interaction	21092	3	7031	F (3, 14) = 1,489	P=0,2605
Row Factor	593,6	1	593,6	F (1, 14) = 0,1257	P=0,7282
Column Factor	29824	3	9941	F (3, 14) = 2,106	P=0,1454
Residual	66092	14	4721		
Difference between row means					
Predicted (LS) mean of Week 5	50,72				
Predicted (LS) mean of Week 12	61,27				
Difference between predicted means	-10,55				
SE of difference	29,75				
95% CI of difference	-74,36 to 53,26				
Within each row, compare columns (simple effects within rows)					
Number of families	2				
Number of comparisons per family	6				
Alpha	0,05				
Tukey's multiple comparisons test	Mean Diff,	95,00% CI of diff,	Significant?	Summary	Adjusted P Value
Week 5					
Negative Control vs. Positive Control	-14,09	-196,4 to 168,2	No	ns	0,9958
Negative Control vs. FMT-CCR	-117,4	-299,7 to 64,93	No	ns	0,2834
Negative Control vs. FMT-Healthy	-54,55	-236,9 to 127,8	No	ns	0,8202
Positive Control vs. FMT-CCR	-103,3	-266,3 to 59,77	No	ns	0,296
Positive Control vs. FMT-Healthy	-40,46	-203,5 to 122,6	No	ns	0,887
FMT-CCR vs. FMT-Healthy	62,83	-100,2 to 225,9	No	ns	0,6837
Week 12					

Negative Control vs. Positive Control	-45,89	-228,2 to 136,4	No	ns	0,8828
Negative Control vs. FMT-CCR	-39,04	-221,3 to 143,3	No	ns	0,9232
Negative Control vs. FMT-Healthy	-140,2	-322,5 to 42,14	No	ns	0,1617
Positive Control vs. FMT-CCR	6,847	-156,2 to 169,9	No	ns	0,9993
Positive Control vs. FMT-Healthy	-94,27	-257,3 to 68,79	No	ns	0,3693
FMT-CCR vs. FMT-Healthy	-101,1	-264,2 to 61,94	No	ns	0,3127

Test details	Mean 1	Mean 2	Mean Diff,	SE of diff,	N1	N2	q	DF
Week 5								
Negative Control vs. Positive Control	4,22	18,31	-14,09	62,72	1	1	0,3177	14
Negative Control vs. FMT-CCR	4,22	121,6	-117,4	62,72	1	1	2,647	14
Negative Control vs. FMT-Healthy	4,22	58,77	-54,55	62,72	1	1	1,23	14
Positive Control vs. FMT-CCR	18,31	121,6	-103,3	56,1	1	1	2,604	14
Positive Control vs. FMT-Healthy	18,31	58,77	-40,46	56,1	1	1	1,02	14
FMT-CCR vs. FMT-Healthy	121,6	58,77	62,83	56,1	1	1	1,584	14
Week 12								
Negative Control vs. Positive Control	5	50,89	-45,89	62,72	1	1	1,035	14
Negative Control vs. FMT-CCR	5	44,04	-39,04	62,72	1	1	0,8803	14
Negative Control vs. FMT-Healthy	5	145,2	-140,2	62,72	1	1	3,16	14
Positive Control vs. FMT-CCR	50,89	44,04	6,847	56,1	1	1	0,1726	14
Positive Control vs. FMT-Healthy	50,89	145,2	-94,27	56,1	1	1	2,376	14
FMT-CCR vs. FMT-Healthy	44,04	145,2	-101,1	56,1	1	1	2,549	14

Supplementary material

S14:

Histopathology of distal Colonic Tissue Week 5

	Positive Control	Positive Control	Positive Control	FMT-CCR	FMT-CCR	FMT-CCR	FMT-Healthy	FMT-Healthy	FMT-Healthy	Negative Control
Epithelial atrophy	0	0	0	0	0	0	0	0	0	0
Glandular atrophy	0	0	0	0	0	0	0	1	0	0
Goblet cell	0	0	0	0	0	0	1	0	0	0
Epithelial hyperplasia	1	0	0	1	0	0	0	0	0	0
Glandular hyperplasia	0	1	1	1	0	1	1	1	0	0
Mitosis	1	1	1	2	2	3	1	1	1	1
Apoptosis	0	0	0	0	1	0	0	0	1	0
Aberrant crypts	0	0	0	1	0	1	1	0	1	0
Epithelial dysplasia	0	0	0	0	0	1	0	0	0	0
Glandular dysplasia	0	0	0	1	0	1	1	1	1	0
Neoplastic proliferation	0	0	3	3	3	2	1	1	3	0
Neoplastic infiltration	1	0	1	1	1	1	1	1	1	1
GALT hyperplasia	2	2	1	1	2	1	1	1	1	0
Erosion	1	0	0	0	0	0	0	0	0	0
Ulceration	1	0	0	0	1	0	0	0	1	0
Pigment	0	0	0	0	0	0	0	0	0	0
Minerals	0	0	0	0	0	0	0	0	0	0
Necrosis	1	0	0	1	1	1	0	0	0	0
Congestion	0	0	0	1	0	1	1	1	1	0
Edema	0	0	0	0	0	0	0	0	1	0
Hemorrhage	0	0	0	0	0	0	0	0	0	0
Thrombosis	0	0	0	0	0	0	0	0	0	0
Fibrin	0	0	0	0	0	0	0	0	0	0
Exocytosis	0	0	0	0	0	0	0	0	0	0
Neutrophils	1	0	1	1	1	1	1	1	1	1
Eosinophils	0	0	0	0	0	0	0	0	0	0
Macrophages	1	1	2	2	2	1	1	1	1	1
Lymphocytes	2	1	2	2	2	1	1	1	1	1
Plasma cells	1	1	2	2	1	1	1	1	1	1
Fibrosis	3	1	3	1	1	1	1	1	1	1
Biological agents	0	0	0	0	0	0	0	0	0	0

Histopathology of Middle Colonic Tissue Week 5

	Positive Control	Positive Control	Positive Control	FMT-CCR	FMT-CCR	FMT-CCR	FMT-Healthy	FMT-Healthy	FMT-Healthy	Negative Control
Epithelial atrophy	0	0	0	0	0	0	0	0	0	0
Glandular atrophy	0	0	0	0	1	0	1	0	1	0
Goblet cell	0	0	0	0	0	0	0	1	0	0
Epithelial hyperplasia	1	1	0	1	0	0	1	0	0	0
Glandular hyperplasia	1	2	1	1	0	1	1	1	0	0

Mitosis	1	1	1	3	2	3	1	1	1	0
Apoptosis	0	0	0	0	0	0	0	0	0	0
Aberrant crypts	1	1	1	1	1	1	1	1	1	0
Epithelial dysplasia	0	0	0	1	0	0	1	0	0	0
Glandular dysplasia	1	1	1	1	1	1	1	1	0	0
Neoplastic proliferation	0	0	1	1	2	2	0	0	1	0
Neoplastic infiltration	1	1	1	1	1	1	0	0	1	1
GALT hyperplasia	3	0	1	3	1	2	1	1	0	0
Erosion	1	0	0	0	0	0	0	0	0	0
Ulceration	0	1	1	0	1	1	0	1	1	0
Pigment	0	0	0	0	0	0	0	0	0	0
Minerals	0	0	0	0	0	0	0	0	0	0
Necrosis	1	1	0	1	1	1	0	1	1	0
Congestion	0	0	0	1	1	1	1	1	0	0
Edema	0	0	0	0	0	0	0	0	0	0
Hemorrhage	0	0	0	0	0	0	0	0	0	0
Thrombosis	0	0	0	0	0	0	0	0	0	0
Fibrin	0	0	0	0	0	0	0	0	0	0
Exocytosis	0	0	0	0	0	0	0	0	0	0
Neutrophils	1	1	1	1	1	1	1	1	1	1
Eosinophils	0	0	0	0	0	0	0	0	0	0
Macrophages	1	2	2	2	2	1	1	1	2	1
Lymphocytes	2	1	1	2	2	2	1	1	1	1
Plasma cells	1	1	1	2	1	2	1	1	1	1
Fibrosis	2	1	2	2	2	1	1	1	1	1
Biological agents	0	0	0	0	0	0	0	0	0	0

Histopathology of distal Colonic Tissue Week 12

	Positive Control	Positive Control	Positive Control	FMT-CCR	FMT-CCR	FMT-CCR	FMT-Healthy	FMT-Healthy	FMT-Healthy	Negative Control
Epithelial atrophy	0	0	0	0	0	0	0	0	0	0
Glandular atrophy	0	0	0	0	0	0	0	0	0	0
Goblet cell	1	1	0	0	0	0	0	0	1	0
Epithelial hyperplasia	1	0	0	0	1	0	0	1	1	0
Glandular hyperplasia	1	1	0	1	1	0	0	1	1	0
Mitosis	1	1	1	3	2	3	2	3	3	0
Apoptosis	0	1	1	0	0	0	1	0	1	0
Aberrant crypts	1	0	0	1	1	0	1	1	1	0
Epithelial dysplasia	0	0	0	0	0	0	0	0	0	0
Glandular dysplasia	1	0	0	0	1	0	0	0	1	0
Neoplastic proliferation	0	1	3	2	3	3	3	3	3	0
Neoplastic infiltration	1	1	1	1	1	1	1	1	1	1
GALT hyperplasia	1	2	1	3	2	1	2	1	0	1
Erosion	0	0	0	0	0	0	0	0	0	0
Ulceration	0	0	0	0	0	0	0	0	0	0

Pigment	0	0	0	0	0	0	0	0	0	0
Minerals	0	0	0	0	0	0	0	0	0	0
Necrosis	0	0	1	1	1	1	1	1	1	0
Congestion	0	1	0	0	1	1	1	1	1	1
Edema	0	0	0	0	0	0	0	0	0	0
Hemorrhage	0	0	0	0	0	0	0	0	0	0
Thrombosis	0	0	0	0	0	0	0	0	0	0
Fibrin	0	0	0	0	0	0	0	0	0	0
Exocytosis	0	0	0	0	0	0	0	0	0	0
Neutrophils	1	1	1	1	1	1	1	1	1	1
Eosinophils	0	0	1	0	0	0	0	0	0	0
Macrophages	1	1	1	1	1	1	1	1	1	1
Lymphocytes	1	1	1	1	1	2	1	1	1	1
Plasma cells	1	1	1	1	1	2	1	1	1	1
Fibrosis	1	1	1	1	1	1	1	1	1	1
Biological agents	0	0	0	0	0	0	0	0	0	0

Histopathology of Middle Colonic Tissue Week 12

	Positive Control	Positive Control	Positive Control	FMT-CCR	FMT-CCR	FMT-CCR	FMT-Healthy	FMT-Healthy	FMT-Healthy	Negative Control
Epithelial atrophy	0	0	0	0	0	0	0	0	0	0
Glandular atrophy	0	0	0	1	0	0	0	0	0	0
Goblet cell	1	0	0	1	0	0	0	0	0	0
Epithelial hyperplasia	0	0	0	0	0	0	0	0	0	0
Glandular hyperplasia	1	0	1	1	1	0	1	1	1	0
Mitosis	1	1	1	1	2	2	1	2	3	0
Apoptosis	0	1	1	0	0	0	1	0	0	0
Aberrant crypts	1	0	0	1	1	0	0	1	1	0
Epithelial dysplasia	0	0	0	0	0	0	0	0	0	0
Glandular dysplasia	1	0	0	1	1	0	0	1	1	0
Neoplastic proliferation	1	2	3	3	3	3	2	3	1	0
Neoplastic infiltration	1	1	1	1	1	1	1	1	1	1
GALT hyperplasia	1	1	1	2	1	3	1	1	1	1
Erosion	0	1	0	0	0	0	0	0	0	0
Ulceration	0	0	0	0	0	0	0	0	0	0
Pigment	0	0	0	0	0	0	0	0	0	0
Minerals	0	0	0	0	0	0	0	0	0	0
Necrosis	0	1	1	1	1	1	1	1	1	0
Congestion	0	0	0	1	1	1	0	1	0	0
Edema	0	0	0	0	0	0	0	0	0	0
Hemorrhage	0	0	0	0	0	0	0	0	0	0
Thrombosis	0	0	0	0	0	0	0	0	0	0
Fibrin	0	0	0	0	0	0	0	0	0	0
Exocytosis	0	0	0	0	0	0	0	0	0	0
Neutrophils	1	1	1	1	1	1	1	1	1	1

Eosinophils	0	0	0	0	0	0	0	0	0	0
Macrophages	1	1	1	1	1	1	1	1	1	1
Lymphocytes	1	1	1	1	1	1	1	1	1	1
Plasma cells	1	1	1	2	1	2	1	1	1	1
Fibrosis	1	1	1	1	1	1	1	1	1	1
Biological agents	0	0	0	0	0	0	0	0	0	0
